# Human pancreatic ductal cells from non-diabetic donors function as non-professional antigen-presenting cells upon inflammatory cytokine exposure

**DOI:** 10.1007/s00125-026-06746-x

**Published:** 2026-05-07

**Authors:** Neslihan Erdem, David Arribas-Layton, Heather N. Zook, Denis O’Meally, Jacob Mares, Janine C. Quijano, Cecile Donohue, Jose A. Ortiz, Kevin Jou, Rupangi C. Vasavada, Enrique Montero, John S. Kaddis, Helena Reijonen, Hsun Teresa Ku

**Affiliations:** 1 Department of Translational Research and Cellular Therapeutics, Arthur Riggs Diabetes and Metabolism Research Institute, City of Hope National Medical Center, Duarte, CA USA; 2 Irell and Manella Graduate School of Biological Sciences, Beckman Research Institute, City of Hope National Medical Center, Duarte, CA USA; 3 Department of Immunology and Theranostics, Arthur Riggs Diabetes and Metabolism Research Institute, City of Hope National Medical Center, Duarte, CA USA; 4 Department of Diabetes and Cancer Discovery Science, Arthur Riggs Diabetes and Metabolism Research Institute, City of Hope National Medical Center, Duarte, CA USA; 5 Department of Diabetes Immunology, Arthur Riggs Diabetes and Metabolism Research Institute, City of Hope National Medical Center, Duarte, CA USA

**Keywords:** Autoreactive CD4^+^ T cells, Cathepsin S, CD40, HLA-DMA, HLA-DMB, HLA-DQ, HLA-DR, Human pancreatic ductal cells, ICAM-1, Inflammatory cytokines

## Abstract

**Aims/hypothesis:**

Type 1 diabetes is an autoimmune disease marked by the destruction of beta cells in pancreatic islets, with an incomplete picture of disease progression and a lack of a definitive cure. A recent finding linked pancreatic ductal cells of type 1 diabetic donors with elevated levels of human leukocyte antigen (HLA) class II molecules; however, the causal relationship and functional significance of this finding remain unknown. Because HLA class II molecules are typically expressed by professional antigen-presenting cells (APCs), this raises the possibility of ductal cells functioning as non-professional APCs. In this study, we test the hypothesis that ductal cells are responsive to type 1 diabetes-associated proinflammatory cytokines, TNF-α, IL-1β and IFN-γ, and can act as non-professional APCs.

**Methods:**

Pancreatic exocrine cells were obtained from cadaveric donors without diabetes following islet removal. Cells were cryopreserved and thawed into a defined culture medium tailored to support ductal cell survival in a 3D suspension culture system. Ductal cells were exposed to various doses of cytokines for 48 h and analysed for gene and protein expression, using quantitative PCR with reverse transcription, bulk RNA-seq, flow cytometry and western blot analyses. Correlation between cytokine response and APC-related gene expression was evaluated using publicly available single-cell RNA-seq datasets from 86 donors. The functional ability of cytokine-treated ductal cells to present an exogenous autoantigen (glutamic acid decarboxylase 65 kDa isoform [GAD65]) to T cells was tested using a GAD65-specific autoreactive CD4^+^ T cell clone (BRI-4.13) isolated from a type 1 diabetic donor.

**Results:**

Within 48 h, a combination of TNF-α, IL-1β and IFN-γ stimulated mRNA and protein expression of HLA class II, co-stimulatory and antigen-processing molecules in non-diabetic ductal cells. Bulk RNA-seq analysis showed that cytokines significantly upregulated biological pathways in ‘antigen processing and presentation’ and ‘type 1 diabetes’. Single-cell RNA-seq analysis revealed a positive correlation between cytokine response and APC gene expression in human pancreatic ductal cells. Cytokine-treated ductal cells pulsed with exogenous GAD65 peptide activated and induced proliferation of BRI-4.13 T cells. Unexpectedly, ~0.9% of KRT19^+^ ductal cells expressed GAD protein endogenously, and 26.6% of KRT19^+^GAD^+^ ductal cells expressed the endocrine marker CHGA.

**Conclusions/interpretation:**

These results demonstrate that non-diseased primary ductal cells respond to TNF-α, IL-1β and IFN-γ by upregulating APC molecules and presenting antigen to autoreactive CD4^+^ T cells. To the best of our knowledge, our results provide the first evidence that non-diabetic human ductal cells can present antigen to T cells, which implicates ductal cells in contributing to type 1 diabetes progression.

**Graphical Abstract:**

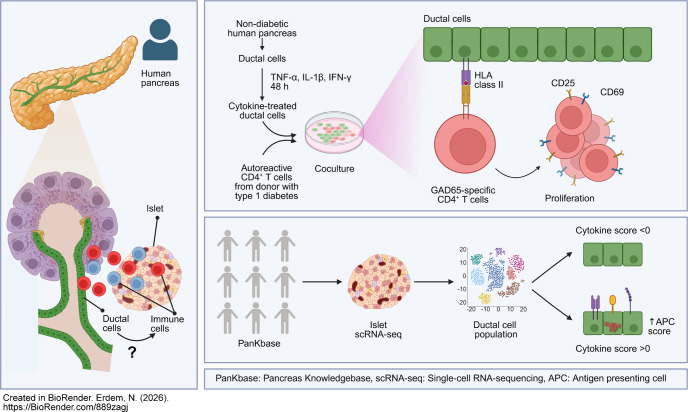

**Supplementary Information:**

The online version contains peer-reviewed but unedited supplementary material available at 10.1007/s00125-026-06746-x.


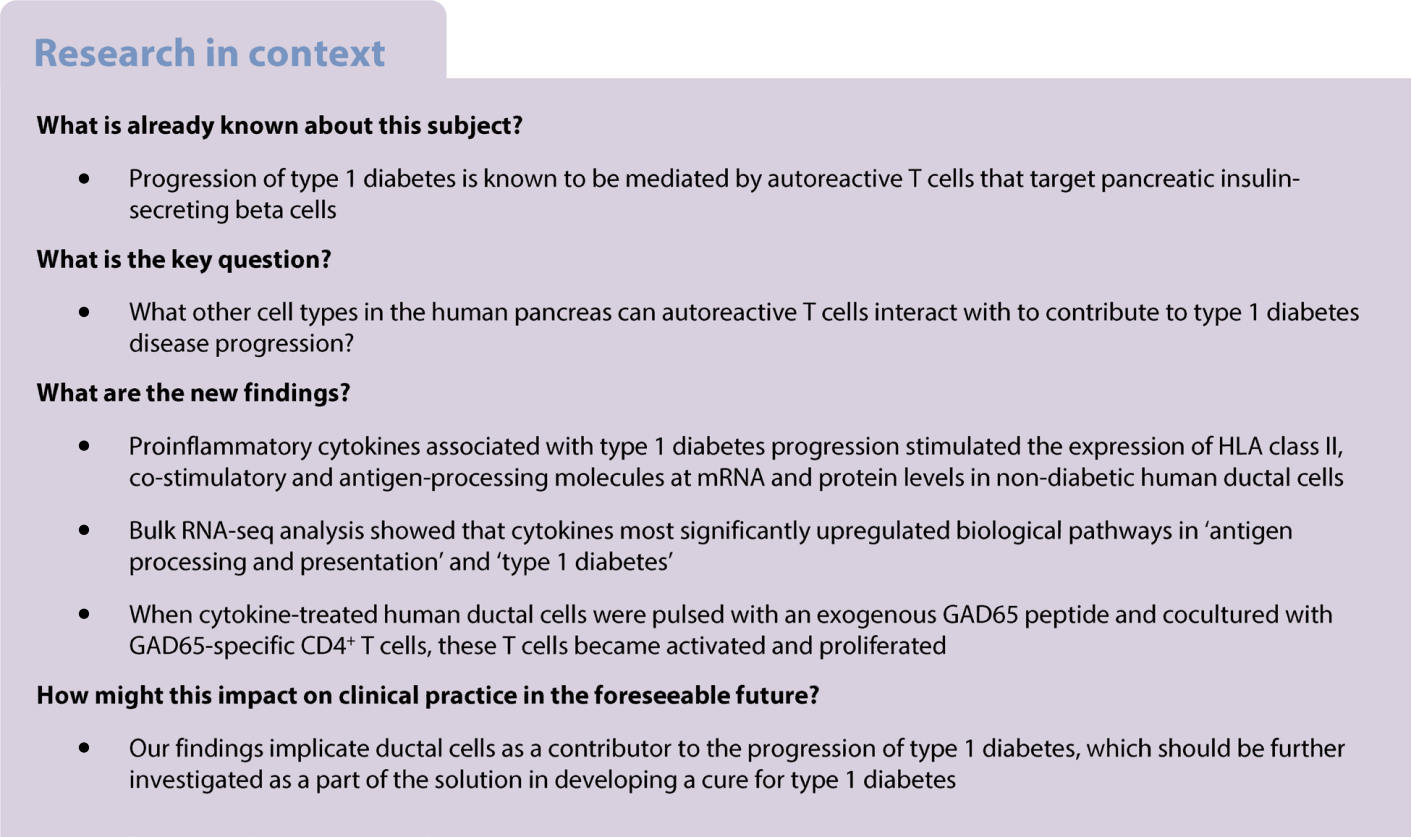


## Introduction

Type 1 diabetes is caused by autoimmune destruction of insulin-producing beta cells in the islets of Langerhans by antigen-specific CD4^+^ and CD8^+^ T cells [1]. Past research has focused on understanding how autoimmune T cells affect beta cells in type 1 diabetes [2]; however, no cure exists to date. CD4^+^ T cells are activated by professional antigen-presenting cells (APCs) expressing human leukocyte antigen (HLA) class II molecules, which present beta cell-derived peptides [3]. Professional APCs include dendritic cells, macrophages and B lymphocytes [3]. Under proinflammatory conditions, beta cells contribute to self-destruction by expressing HLA class II molecules that present self-peptides, and autoreactive CD4^+^ T cells targeting these self-antigens can be found in the peripheral blood or islets [4–6]. Type 1 diabetes disease susceptibility is strongly linked to specific HLA class II haplotypes, especially HLA-DRB1*04-DQA1*03-DQB1*03:02 (DR4-DQ8) and HLA-DRB1*03:01-DQB1*05:01-DQB1*02:01 (DR3-DQ2) [1, 2, 7]. These findings implicate HLA class II-expressing cells in type 1 diabetes progression. However, the contribution of other pancreatic cell types in the type 1 diabetes disease mechanism remains underexplored.

Pancreatic exocrine ductal cells form the epithelial lining that transports digestive enzymes secreted by acinar cells into the duodenum. In type 1 diabetes, increased T cell infiltration in the exocrine pancreas suggests immune involvement beyond the islets [8]. Epithelial cells, such as those in the gut, can function as non-professional APCs by upregulating HLA class II and promoting T cell proliferation [9, 10]. Recently, pancreatic ductal cells from type 1 diabetes donors have been linked to HLA-DR expression, suggesting a role in antigen presentation [11–14]. However, whether ductal cells from non-diabetic donors respond to type 1 diabetes-associated proinflammatory cytokines (TNF-α, IL-1β and IFN-γ) [15] and present antigens to T cells remains unclear. In this study, we therefore tested the hypothesis that human exocrine ductal cells are responsive to TNF-α, IL-1β and IFN-γ, and can function as non-professional APCs.

## Methods

### Supplementary methods

Additional details for all methods are provided in the electronic supplementary material (ESM).

### Primary human pancreatic exocrine cells

Human pancreases from deceased donors without diabetes (ESM Tables 1, 2; ESM Human Islets Checklist) were obtained through the Southern California Islet Cell Resource Center (City of Hope) for islet isolation [16]. De-identified pancreatic exocrine tissues were provided under IRB Protocol No. 23728 (Non-Human Subjects Research). Exocrine tissue dissociation, cryopreservation and 3D suspension culture were performed as described [17], using specific media for ductal cell survival [17, 18] (ESM Table 3).

### Primary human CD4^+^ T cell clones

Two previously isolated CD4^+^ T cell clones from individuals with type 1 diabetes were used: BRI-4.13 (glutamic acid decarboxylase 65 kDa isoform [GAD65]-specific, HLA-DRB1*04:01-restricted) and BRI-5.325 (GAD65-specific, HLA-DRB1*04:04-restricted) [5]. A haemagglutinin (HA)-specific (HLA-DRB1*04:04-restricted) CD4^+^ T cell clone was isolated from a healthy HLA-DRB1*04:04, HLA-DRB1*03:01 donor.

### Cell lines

Human pancreatic ductal epithelial cells (HPDECs) (AddexBio, cat. no. T0018001) and B lymphoblastoid cell line (B-LCL) BSM (Sigma, cat. no. 88052032) were cultured per manufacturer instructions.

### Proinflammatory cytokine treatment

Ductal cells were treated for 48 h with TNF-α, IL-1β and IFN-γ (doses indicated in ESM Table 4).

### qRT-PCR

Quantitative PCR with reverse transcription (qRT-PCR) was performed using TaqMan probes (Thermo Fisher Scientific, ESM Table 5) with *ACTB* as reference and two or three technical replicates per run [19].

### Bulk RNA-seq

Sequencing was conducted on the NovaSeq X Series platform. Ranked lists of genes according to their log_2_ fold change (FC) and *p* values are provided in ESM Tables 6 and 7. Significantly enriched pathways along with their associated genes are presented in ESM Tables 8–19.

### scRNA-seq analysis of ductal cells

Single-cell RNA-seq (scRNA-seq) data from human pancreatic islets were obtained from the Human Pancreas Analysis Program (HPAP) and published studies via the Pancreas Knowledgebase (PanKbase) repository [11, 20–22]. The final analysis cohort comprised 86 donors (20,605 ductal cells) including individuals with no diabetes mellitus (NODM), autoantibody-positive individuals (AABP) and individuals with type 1 diabetes. Donor characteristics are provided in ESM Table 20. Cells from experimental perturbation conditions were excluded; only untreated or vehicle control cells from donors with curated diabetes status, age and sex were retained.

### Flow cytometry

For data in Fig. 2 and ESM Fig. 3, ductal cells were incubated with fluorophore-conjugated or biotin-conjugated monoclonal antibodies (ESM Table 21) in 100 μl of flow cytometry buffer at 4°C for 30 min in the dark, and with streptavidin-labelled allophycocyanin (Miltenyi, cat. no. 130-106-792) for 30 min at 4°C (ESM Table 21). After staining, cells were washed twice, centrifuged at 300 *g* for 5 min and resuspended in 200 μl of flow cytometry buffer.

For coculture experiments, cells collected at indicated time points were washed and stained with BD Via-Probe Cell Viability Solution (BD Biosciences, cat. no. 555815) and fluorochrome-conjugated antibodies for 20 min at 4°C in the dark (ESM Table 21). After staining, cells were washed twice, centrifuged at 300 *g* for 5 min and resuspended in 200 μl of flow cytometry buffer. For proliferation assay, CD4^+^ T cell clones were stained with CellTrace carboxyfluorescein succinimidyl ester (CFSE) (Thermo Fisher Scientific, cat. no. C34554) at 2.5 µmol/l prior to coculture, and proliferation tracking was conducted according to the manufacturer’s protocol.

### Western blot

Cells were lysed in RIPA buffer with protease and phosphatase inhibitors. Blots were quantified by ImageJ [23], and normalised to β-actin. Antibodies are listed in ESM Table 21.

### Coculture experiments

Ductal cells from donors with HLA-DRB1*04:01 (matching BRI-4.13) or HLA-DRB1*04:04 haplotype (matching BRI-5.325 and HA-specific CD4^+^ T cell clones) were used for coculture assays (ESM Table 22). CD4^+^ T cells were cocultured with matched ductal cells or APCs at a 1:5 ratio (100,000 T cells to 500,000 APCs) or stimulated with plate-bound anti-CD3 antibodies at 1 µg/ml in 48-well plates (1 ml per well). Cocultures were maintained for up to 5 days at 37°C and 5% CO₂.

### Immunofluorescence staining

Formalin-fixed, paraffin-embedded pancreas slides were stained as described [19], and imaged on a Zeiss LSM 880 confocal microscope. Antibodies are listed in ESM Table 21.

### Statistical analysis

Statistical analyses and plots were generated using GraphPad Prism v9.5 or v10. Data are presented as mean ± SEM or mean ± SD (see figure legends). Significance levels between two groups were determined using unpaired two-tailed *t* test and those between three or more groups were determined using ordinary one-way ANOVA. Significance was set at *p*<0.05 and denoted as *p<0.05, **p<0.01, ***p<0.001. Additional details are in figure legends.

## Results

### Proinflammatory cytokines increase HLA class II genes in human pancreatic ductal cells in a dose-dependent manner

To study primary human ductal cells from donors without diabetes and other chronic diseases, our laboratory developed a 3D suspension culture system that supports ductal cell survival, but not acinar or endocrine cells, in serum-free medium containing defined growth factors [17]. scRNA-seq of day 3 and day 7 suspension cells from cryopreserved and freshly dissociated exocrine tissues revealed that approximately 83.7% of all cells are ductal cells [17]. In this study, further analysis showed that cryopreserved samples were more enriched for ductal cells (89.1% and 90.6%) than fresh samples (ESM Fig. 1a). Therefore, only cryopreserved exocrine samples were used in subsequent experiments. Overall, donors were 37.18±10.13 years old, had a BMI of 25.48±4.54 kg/m^2^ and had HbA_1c_ of 5.16±0.32% (ESM Tables 1, 2).

**Fig. 1:**
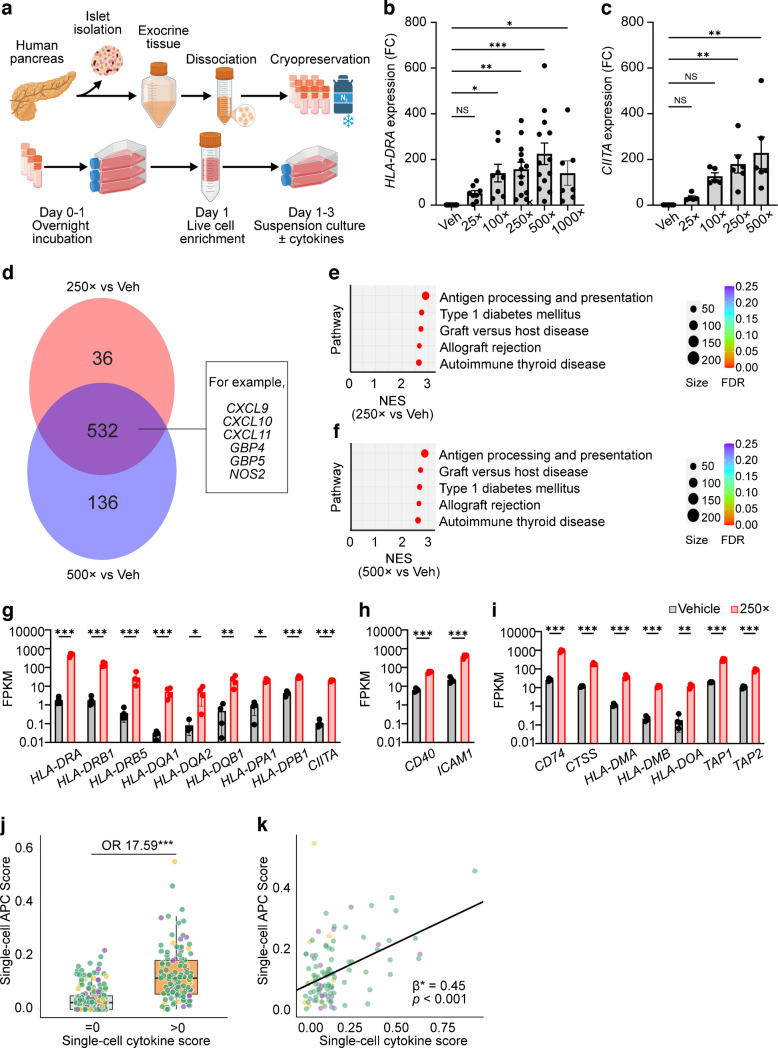
Type 1 diabetes-associated proinflammatory cytokines induce the expression of genes involved in antigen processing, presentation and co-stimulation in ductal cells of non-diabetic human pancreases. (**a**) Experimental workflow of the 3D suspension culture system for human ductal cells. Cells were treated for 48 h with graded doses of cytokines (see ESM Table 4). Created with BioRender.com. Erdem, N. (2026). https://BioRender.com/ozzlpri (**b**, **c**) Conventional qRT-PCR analysis of *HLA-DRA* (**b**) and *CIITA* (**c**). *N*=5–13 biological replicates from 3–10 unique donors. Each dot represents a biological replicate, with value as the mean of *n*=3 technical replicates. Data represent mean ± SEM. One-way ANOVA with Dunnet’s test was used for statistical analysis. Each experimental group was compared with vehicle control, with significance indicated as *p<0.05, **p<0.01, ***p<0.001 and *p*>0.05 not significant (NS). (**d**) Venn diagram showing DEGs with FDR<0.05 and log_2_FC>1, comparing 250× vs vehicle (red) and 500× vs vehicle (blue). *N*=4 donors. (**e**, **f**) GSEA comparing 250× vs vehicle (**e**) and 500× vs vehicle (**f**), using the KEGG molecular signature database. *N*=4 donors. (**g**–**i**) Expression levels of FPKM of markers for HLA class II (**g**), co-stimulatory (**h**) and antigen processing and loading molecules (**i**). *N*=4 donors. Data represent mean ± SD. Multiple paired *t* test was used for statistical analysis, with significance indicated as *p<0.05, **p<0.01, ***p<0.001. (**j**) Cytokine response correlates with APC gene score in scRNA-seq analysis. APC score in individual ductal cells is stratified by cytokine response score (*n*=20,494 cells for cytokine score=0; *n*=111 cells for cytokine score > 0). The box-and-whisker plot shows median and interquartile range (IQR; box) and 1.5 × IQR (whiskers). A random 1% sample of cytokine score=0 cells is shown to avoid overplotting. The binary component of a mixed-effects hurdle model (ESM Methods) confirmed that cytokine-positive cells had >17-fold higher odds of expressing the APC programme (OR 17.59 [95% CI 5.53, 56], *p*<0.001). (**k**) Dose–response of cytokine–APC scores. Double-positive cells with both APC score > 0 and cytokine score > 0 (*n*=108 cells from 33 donors) were used for the hurdle model Part 2 (positive component). Among these cells, cytokine response magnitude predicts APC magnitude (β*=0.45 [95% CI 0.28–0.62], *p*<0.001) (ESM Methods). Panels (**j**, **k**): points coloured by diabetes status (NODM, green; AABP, yellow; type 1 diabetes, purple). FDR, false-discovery rate; FPKM, fragments per kb of transcript per million mapped reads; KEGG, Kyoto Encyclopedia of Genes and Genomes; NES, normalised expression score; Veh, Vehicle

After thawing cryopreserved exocrine cells and overnight culturing in 3D suspension (ESM Table 3), live ductal cells were enriched by density gradient centrifugation [17, 18]. Day 1 cells were then treated for 48 h with graded doses of type 1 diabetes-associated cytokines (ESM Table 4) TNF-α, IL-1β and IFN-γ [24, 25] (Fig. 1a). Notably, a ‘1000×’ dose (1000 IU/ml TNF-α, 100 IU/ml IL-1β and 1000 IU/ml IFN-γ) has been shown to induce cell death in isolated human islets [24].

Increased ductal expression of HLA class II molecules has been reported in individuals with type 1 diabetes [11]. Within 48 h, *HLA-DRA* expression was increased in a dose-dependent manner in non-diabetic ductal cells, beginning at 100× and plateauing at 500× (Fig. 1b). The HLA class II master regulator *CIITA* [26] was increased at 250× and 500×, but not at lower doses (Fig. 1c); therefore 250× and 500× doses were selected for the next experiment.

### RNA-seq analysis reveals upregulation of pathways involved in ‘antigen processing and presentation’ and ‘type 1 diabetes’ in cytokine-treated ductal cells

To evaluate global transcriptomics, we treated human ductal cells with cytokines and vehicle control for 48 h and performed bulk RNA-seq (*N*=4 donors). Multidimensional scaling (MDS) analysis showed a clear separation of the 250× and 500× conditions from the vehicle control (ESM Fig. 1b), demonstrating a robust effect of cytokines. Overall, 1010 differentially expressed genes (DEGs) were identified by comparing 250× vs vehicle, and 1346 DEGs in the 500× vs vehicle comparison (ESM Fig. 1c, d, ESM Tables 6, 7).

Comparison of upregulated DEGs from 250× and 500× treatments showed 76% overlap (532 of 704 unique genes) (Fig. 1d). Similarly, 56% of the downregulated DEGs overlapped (400 of 720 unique genes) (ESM Fig. 1c, d, ESM Tables 6, 7). These results demonstrate commonality in DEGs induced by 250× and 500× cytokine doses, compared with vehicle control.

To gain further insight into the biological pathways induced by cytokines in ductal cells, gene set enrichment analysis (GSEA) was conducted, which revealed significant upregulation of pathways associated with 'antigen processing and presentation' and 'type 1 diabetes' in both 250× and 500× conditions (Fig. 1e, f, ESM Fig. 1e–h). Other commonly upregulated pathways included ‘graft vs host disease’, ‘allograft rejection’, ‘proteasome’, ‘NOD like receptor signalling pathway’ and ‘response to virus’ (ESM Fig. 1e–j), all of which are important for innate and adaptive immunity. Considering the similarity of the DEGs by both 250× and 500× cytokines, we proceeded with using the lower dose (250×) in subsequent experiments.

Antigen processing and presentation to T cells involves antigen uptake, proteolysis, loading onto HLA molecules and display at the cell surface [27]. In addition to HLA molecules, T cells also require a second signal, the co-stimulatory molecules, for activation [28]. In ductal cells treated with 250× cytokines vs vehicle control, bulk RNA-seq analysis revealed upregulation of molecules for HLA class II (*HLA-DRA*, *HLA-DRB1*, *HLA-DRB5*, *HLA-DQA1*, *HLA-DQA2*, *HLA-DQB1*, *HLA-PA1*, *HLA-PDB1*, *CIITA*) (Fig. 1g), co-stimulation (*CD40*, *ICAM1*) (Fig. 1h) and antigen processing and loading (*CD74*, *CTSS*, *HLA-DMA*, *HLA-DMB*, *HLA-DOA*, *TAP1*, *TAP2*) (Fig. 1i), demonstrating that cytokines induce the expression of genes involved in antigen processing and presentation in ductal cells.

### Publicly available scRNA-seq datasets reveal a positive correlation between cytokine response and APC genes in ductal cells

Next, we analysed publicly available scRNA-seq datasets [11, 20–22] (ESM Table 20) to test whether individual ductal cells with a higher inflammatory score express higher levels of APC-related genes. Cytokine response score was based on six upregulated genes (*CXCL9*, *CXCL10*, *CXCL11*, *GBP4*, *GBP5*, *NOS2*) derived from the above bulk RNA-seq (ESM Table 6), whereas the APC score was from the 18 upregulated genes shown in Fig. 1g–i.

We found that single ductal cells with detectable cytokine responses (cytokine score >0) were more likely to express APC genes (OR 17.59 [95% CI 5.53, 56], *p*<0.001), and this activation occurred regardless of diabetes status (Fig. 1j). Among cells with APC score >0 and cytokine score >0 (*n*=108 cells from 33 donors), the magnitude of the cytokine response increased with APC expression (Fig. 1k). Further analysis suggested that donor-level factors modulate the strength of the cytokine response–APC gene association; however, the association remained positive for all individual donors examined (ESM Fig. 2a).

### Cytokine-treated ductal cells upregulate the expression of proteins involved in antigen presentation and antigen processing and loading

To determine protein expression on ductal cell surfaces, we performed multiplex flow cytometry analysis. Ductal cells were identified by CD133 (also known as PROM1), a known surface marker for pancreatic ductal cells [29, 30] (ESM Fig. 3a, b). The frequency of CD133^+^ ductal cells was 75–94% and 73–93% in the vehicle-treated and 250× cytokine-treated groups, respectively (ESM Fig. 3c), with no significant difference between groups (*N*=12 biological replicates from 11 unique donors). Similarly, the mean fluorescence intensity (MFI) of CD133 expression remained unchanged between treatments (ESM Fig. 3d). These results demonstrate that cytokine exposure does not change CD133 expression in ductal cells.

**Fig. 2:**
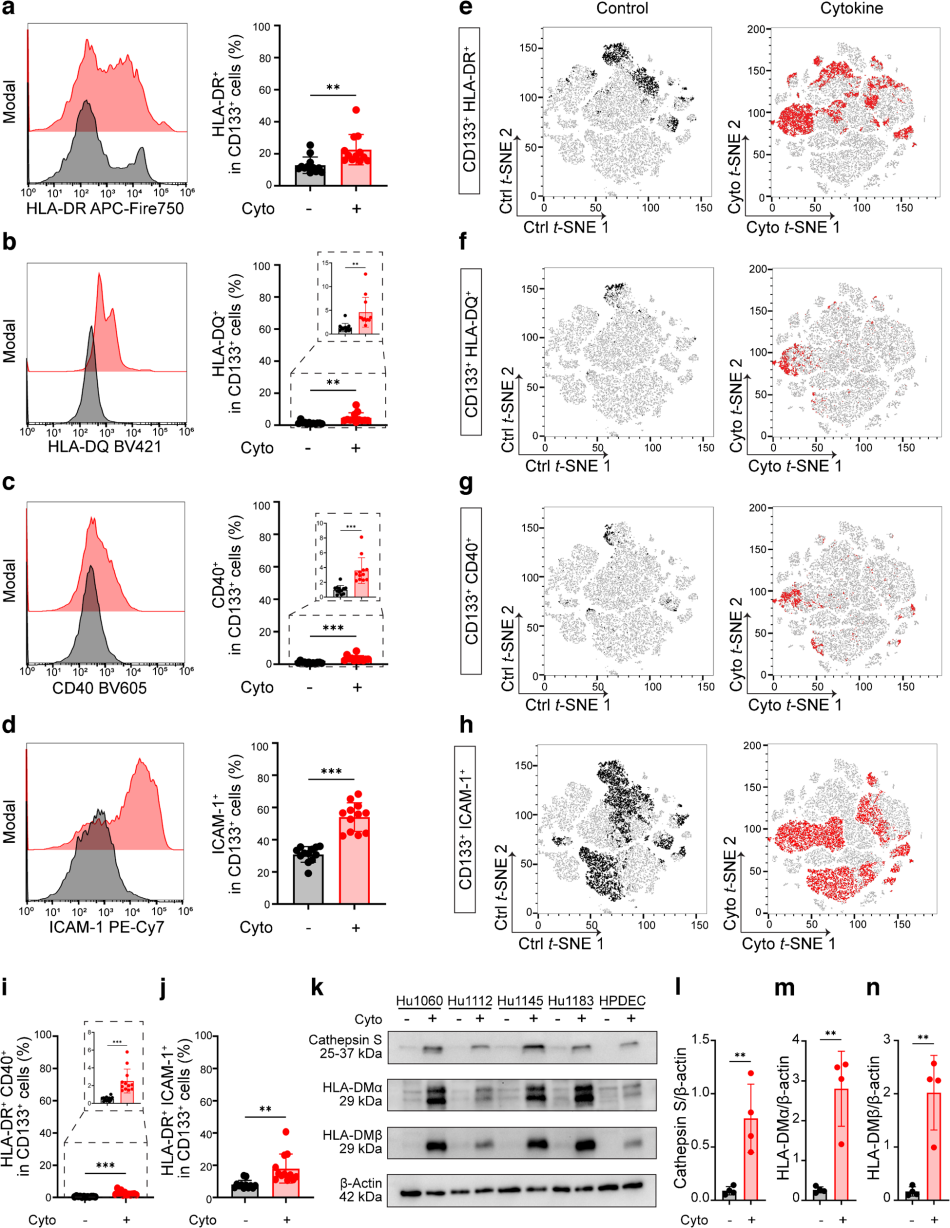
Proteins involved in HLA class II, co-stimulation and antigen processing and loading are upregulated in response to cytokines. (**a**–**d**) Representative histograms and quantification of percentage of HLA-DR^+^ (**a**), HLA-DQ^+^ (**b**), CD40^+^ (**c**) or ICAM-1^+^ (**d**) cells among CD133^+^ ductal cells. *N*=12 biological replicates from 11 unique donors. Each dot represents a biological replicate, with value as the mean of *n*=1–3 technical replicates. Data represent mean ± SD. Unpaired *t* test was used for statistical analysis, with significance indicated as **p<0.01, ***p<0.001. (**e**–**h**) *t*-SNE analysis of multiplex flow cytometry. Each dot represents a single cell. Marker-positive cells from the vehicle control group are shown in black, and those from cytokine-treated groups in red. (**i**, **j**) Quantification of percentage of HLA-DR^+^CD40^+^ (**i**) and HLA-DR^+^ICAM-1^+^ (**j**) double-positive cells among CD133^+^ cells. *N*=12 biological replicates from 11 unique donors. Each dot represents a biological replicate, with value as the mean of *n*=1–3 technical replicates. Data represent mean ± SD. Unpaired *t* test was used for statistical analysis, with significance indicated as **p<0.01, ***p<0.001. (**k**) Western blot analysis of cathepsin S, HLA-DMα and HLA-DMβ in ductal cells treated with vehicle or cytokines for 48 h (*N*=4 donors). HPDEC is an immortalised human ductal cell line. (**l**–**n**) Densitometry analyses of cathepsin S (**l**), HLA-DMα (**m**) and HLA-DMβ (**n**) in vehicle- and cytokine-treated primary human ductal cells. Densitometry values were normalised to β-actin housekeeping protein. *N*=4 donors. Data represent mean ± SD. Unpaired *t* test was used for statistical analysis, with significance indicated as **p<0.01

A higher percentage of CD133^+^ ductal cells expressed HLA-DR and HLA-DQ after cytokine treatment compared with vehicle controls (Fig. 2a, b). Cytokine treatment also increased the proportion of CD133^+^ ductal cells expressing co-stimulatory molecules CD40 and ICAM1 (Fig. 2c,d), but not CD80 and CD86 (ESM Fig. 3e, f), consistent with bulk RNA-seq analysis (Fig. 1h, ESM Table 6). These results demonstrate that, compared with vehicle control, cytokines induce surface expression of HLA class II and co-stimulatory proteins in a subset of ductal cells.

To determine whether the above-mentioned surface proteins were expressed simultaneously on a cell, we used *t*-distributed stochastic neighbour embedding (*t*-SNE) analysis on the multiplex flow cytometry data, which revealed that a subgroup of CD133^+^ ductal cells expressed both HLA class II and co-stimulatory molecules (Fig. 2e–h); co-expression of these molecules is essential for APC function [28]. Importantly, cytokine-treated ductal cells showed a higher percentage of HLA-DR^+^ICAM-1^+^ and HLA-DR^+^CD40^+^ double-positive cells compared with vehicle-treated cells (Fig. 2i, j), demonstrating that multiple cell-surface proteins are induced on the same ductal cells.

To confirm intracellular changes in antigen-processing and loading proteins [27], we conducted western blot analysis (*N*=4 donors). Cathepsin S, HLA-DMα and HLA-DMβ were increased 8.5-fold, 10.6-fold and 12.1-fold in cytokine-treated ductal cells, compared with vehicle control, respectively (Fig. 2k–n, ESM Fig. 4a–d). Similar results were observed in HPDECs (Fig. 2k–n, ESM Fig. 4a–d), an immortalised human pancreatic ductal cell line [31]. Together, these results demonstrate that cytokines, compared with vehicle controls, induce intracellular proteins involved in antigen processing in ductal cells.

### Cytokine-treated ductal cells pulsed with an exogenous GAD65 peptide activate a GAD65-specific CD4^+^ T cell clone

Upregulation of HLA class II and co-stimulatory molecules on cytokine-treated ductal cells (Fig. 2) suggests potential antigen presentation and CD4^+^ T cell activation. GAD65 is a well-established autoantigen in type 1 diabetes [5, 32, 33]. We previously isolated, expanded and cryopreserved a GAD65 555–567-specific autoreactive CD4^+^ T cell clone (BRI-4.13) from the peripheral blood of an individual with type 1 diabetes [5]. BRI-4.13 responds to wild-type GAD65 (NFFRMVISNPAAT; residues 555–567) [5]. However, to maximise HLA class II binding and T cell stimulation, in subsequent studies we used a 13-amino-acid peptide (NFIRMVISNPAAT) containing a single phenylalanine (F) to isoleucine (I) substitution at position 557 [33].

**Fig. 3:**
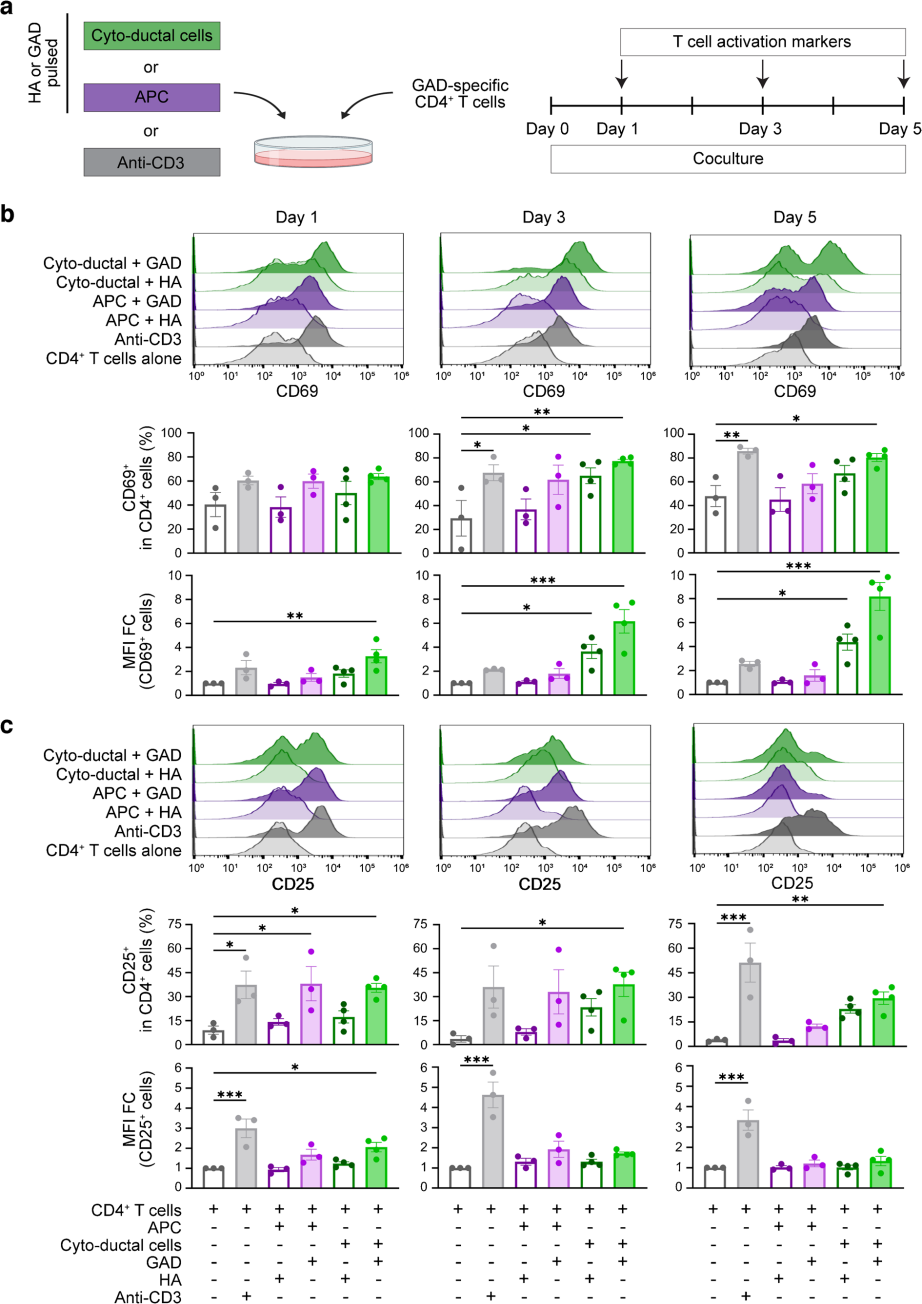
Cytokine-treated ductal cells (cyto-ductal cells) pulsed with a GAD65 antigen peptide stimulate activation markers on GAD65-specific CD4^+^ T cells. (**a**) Experimental workflow for BRI-4.13 T cell activation assay. Created with BioRender.com. Erdem, N. (2026) https://BioRender.com/8exvpm2 (**b**, **c**) Top, middle and lower panels indicate representative histograms, percentage of CD4^+^ cells expressing the designated marker and the FC of MFI of the designated marker in an experimental group relative to the CD4^+^ T cells alone group, respectively. The colour scheme indicates cultured BRI-4.13 CD4^+^ T cells alone (open grey), stimulated with anti-CD3 (filled grey), cocultured with HA-pulsed (HA306 peptide, PKYVKQNTLKLAT, open purple) or GAD65-pulsed (GAD65 557I, NFIRMVISNPAAT, filled purple) BSM APCs, and cocultured with HA-pulsed (HA306 peptide, PKYVKQNTLKLAT, open green) or GAD65-pulsed cytokine-treated ductal cells (GAD65 557I, NFIRMVISNPAAT, filled green). End points were analysed at days 1, 3 and 5 post coculture as shown in the left, middle and right panels, respectively. *N*=3–4 biological replicates of CD4^+^ T cells that were cocultured with ductal cells from three unique donors. Each dot represents a biological replicate, with value as the mean of *n*=2 technical replicates. Data represent mean ± SEM. One-way ANOVA with Dunnett’s test was used for statistical analysis. Comparisons of each experimental group were made with CD4^+^ T cell only control. Significance is indicated as *p<0.05, **p<0.01, ***p<0.001

To assess APC function, cytokine-treated ductal cells were pulsed with the GAD65 peptide (NFIRMVISNPAAT) and cocultured with BRI-4.13 CD4^+^ T cells (Fig. 3a). Because BRI-4.13 is HLA-DRB1*04:01-restricted [5], ductal cells were obtained from donors with this haplotype selected from our exocrine biobank of >70 unique donors. Positive controls were T cells treated with anti-CD3 antibodies or GAD65 peptide-pulsed BSM cells, an HLA-DRB1*04:01^+^ Epstein–Barr virus-transformed B-LCL capable of antigen presentation [34, 35]. An HLA-DRB1*04:01-binding influenza HA peptide [36] was used as an irrelevant antigen negative control (Fig. 3a).

**Fig. 4:**
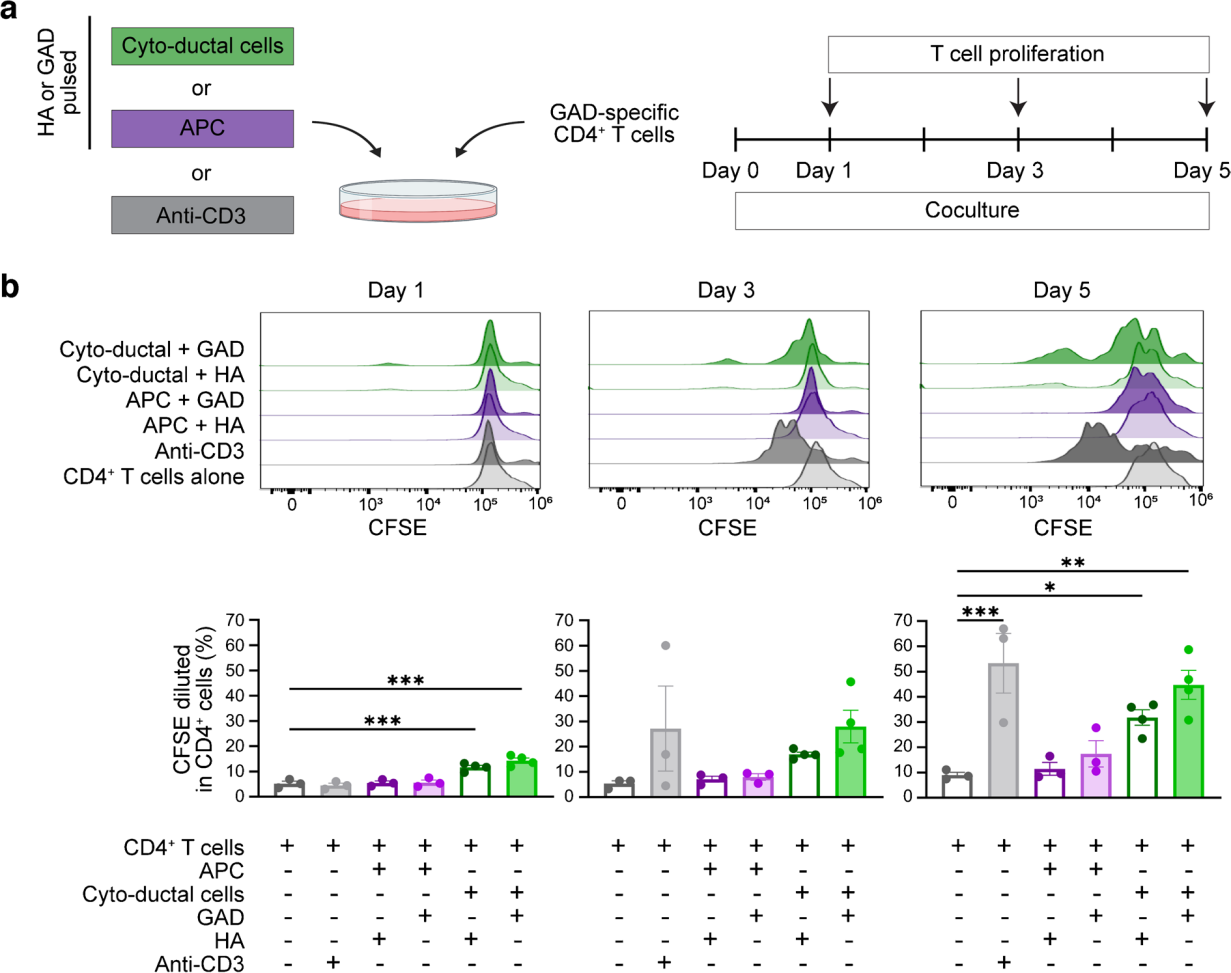
Proliferation of GAD65-specific CD4^+^ T cells is increased by cytokine-treated ductal cells (cyto-ductal cells) pulsed with a GAD65 antigen peptide. Created with BioRender.com. Erdem, N. (2026) https://BioRender.com/m0nhvay (**a**) Experimental workflow for BRI-4.13 T cell proliferation assay. (**b**) Representative histograms and quantification of percentage of CFSE-diluted CD4^+^ T cells. The colour scheme and end points are the same as indicated in Fig. 3. *N*=3–4 biological replicates of CD4^+^ T cells that were cocultured with ductal cells from three unique donors. Each dot represents a biological replicate, with value as the mean of *n*=2 technical replicates. Data represent mean ± SEM. One-way ANOVA with Dunnett’s test was used for statistical analysis. Comparisons of each experimental group were made with CD4^+^ T cell only control. Significance is indicated as *p<0.05, **p<0.01, ***p<0.001

Flow cytometry showed that stimulation with GAD65-pulsed, cytokine-treated ductal cells increased the percentage of BRI-4.13 CD4^+^ T cells expressing CD69 (a T cell activation marker) at days 3 and 5, compared with untreated control T cells (Fig. 3b, ESM Fig. 5a,b). CD69 MFI on CD69^+^CD4^+^ T cells was also higher at days 1, 3 and 5 (Fig. 3b). The percentage of CD4^+^ T cells expressing CD25 (also known as IL2RA), another T cell activation marker, also increased at days 1, 3 and 5 (Fig. 3c). T cell proliferation, assessed by CFSE dilution (Fig. 4a, ESM Fig. 5c), was enhanced at days 1 and 5 (Fig. 4b). Overall, these results demonstrate that cytokine-treated ductal cells can present exogeneous GAD peptide and induce activation and proliferation of an autoreactive CD4^+^ T cell clone.

Using the same dataset, we directly compared ductal cells with BSM APCs. Upon coculture with GAD-pulsed cells, T cells exposed to ductal cells showed similar or higher percentages of CD69 and CD25 expression, as well as higher CD69 MFI in CD69^+^CD4^+^ T cells and CD25 MFI in CD25^+^CD4^+^ T cells, compared with BSM cells (ESM Fig. 6a,b). Moreover, GAD-pulsed cytokine-treated ductal cells induced greater T cell proliferation at all timepoints compared with BSM cells (ESM Fig. 6c). These results demonstrate that ductal cells are equally as effective as or more effective than BSM APCs at stimulating autoreactive CD4^+^ T cells.

### Cytokine-treated ductal cells without exogenous GAD65 peptide activate a GAD65-specific CD4^+^ T cell clone

Surprisingly, cytokine-treated ductal cells pulsed with the irrelevant HA peptide also stimulated GAD65-specific BRI-4.13 CD4^+^ T cells, compared with untreated BRI-4.13 cells, leading to increased CD69^+^ cell frequency at day 3, higher CD69 expression at days 3 and 5, and enhanced proliferation at days 1 and 5 (Figs 3b, 4b). However, these responses were weaker than those induced by GAD65-pulsed cytokine-treated ductal cells.

Direct comparison with BSM APCs showed that HA-pulsed, cytokine-treated ductal cells induced higher percentages of CD69⁺ T cells at day 3 (ESM Fig. 6d) and CD25⁺ T cells at day 5 (ESM Fig. 6e), greater T cell proliferation at days 1, 3 and 5 (ESM Fig. 6f) and enhanced CD69 MFI on days 3 and 5 (ESM Fig. 6d). These results demonstrate again that cytokine-treated ductal cells are equally as effective as or more effective than BSM APCs even in the absence of exogenous GAD.

### Cytokine-treated ductal cells pulsed with an exogenous HA peptide activate an HA-specific CD4^+^ T cell clone

To further assess antigen specificity, we cocultured an HA-specific CD4^+^ T cell clone with cytokine-treated ductal cells pulsed with HA or GAD antigen (ESM Fig. 7a). HA-pulsed, cytokine-treated ductal cells induced a higher percentage of CD69^+^ cells at day 3 and greater CD69 MFI at days 3 and 5 compared with GAD-pulsed cytokine-treated ductal cells (ESM Fig. 7b). CD25^+^ frequency, CD25 MFI (ESM Fig. 7c) and T cell proliferation (ESM Fig. 7d) were also increased at days 1, 3 and 5. Together, these results demonstrate that cytokine-treated ductal cells activate HA-specific CD4^+^ T cells in an antigen-dependent manner, and their antigen-presenting function is not limited to GAD65-specific CD4^+^ T cells.

### Cytokine exposure enhances APC function of ductal cells

Control ductal cells also express HLA class II and co-stimulatory molecules (Fig. 2a–d). To evaluate their antigen-presenting capabilities, BRI-4.13 CD4^+^ T cells were cocultured with control- or cytokine-treated ductal cells that were pulsed with GAD or HA (ESM Fig. 8a). Compared with unstimulated T cells, cytokine-treated ductal cells increased T cell proliferation at days 1, 3 and 5, whereas control ductal cells had no effect at days 3 and 5 (ESM Fig. 8b). These results indicate that cytokine exposure increases efficiency of ductal cell antigen presentation.

### GAD65/67 protein is expressed endogenously by pancreatic ductal cells

The finding that cytokine-treated ductal cells activated a GAD65-specific CD4^+^ T cell clone without exogenous peptide (Figs 3, 4) suggests endogenous GAD expression. The peptide recognised by BRI-4.13 (NFFRMVISNPAAT) is identical in both GAD65 and glutamic acid decarboxylase 67 kDa (GAD67) isoforms. While GAD65, encoded by *GAD2*, is mostly expressed in endocrine cells and not ductal cells [37], publicly available scRNA-seq data show a subset of ductal cells associated with isolated islets express *GAD1* [38], encoding GAD67 (73% C-terminal homology with GAD65 [39]). Consistently, our previous scRNA-seq of freshly isolated ductal cells from exocrine tissue after islet depletion also detected *GAD1* in some ductal cells (ESM Fig. 9a) [30].

**Fig. 5:**
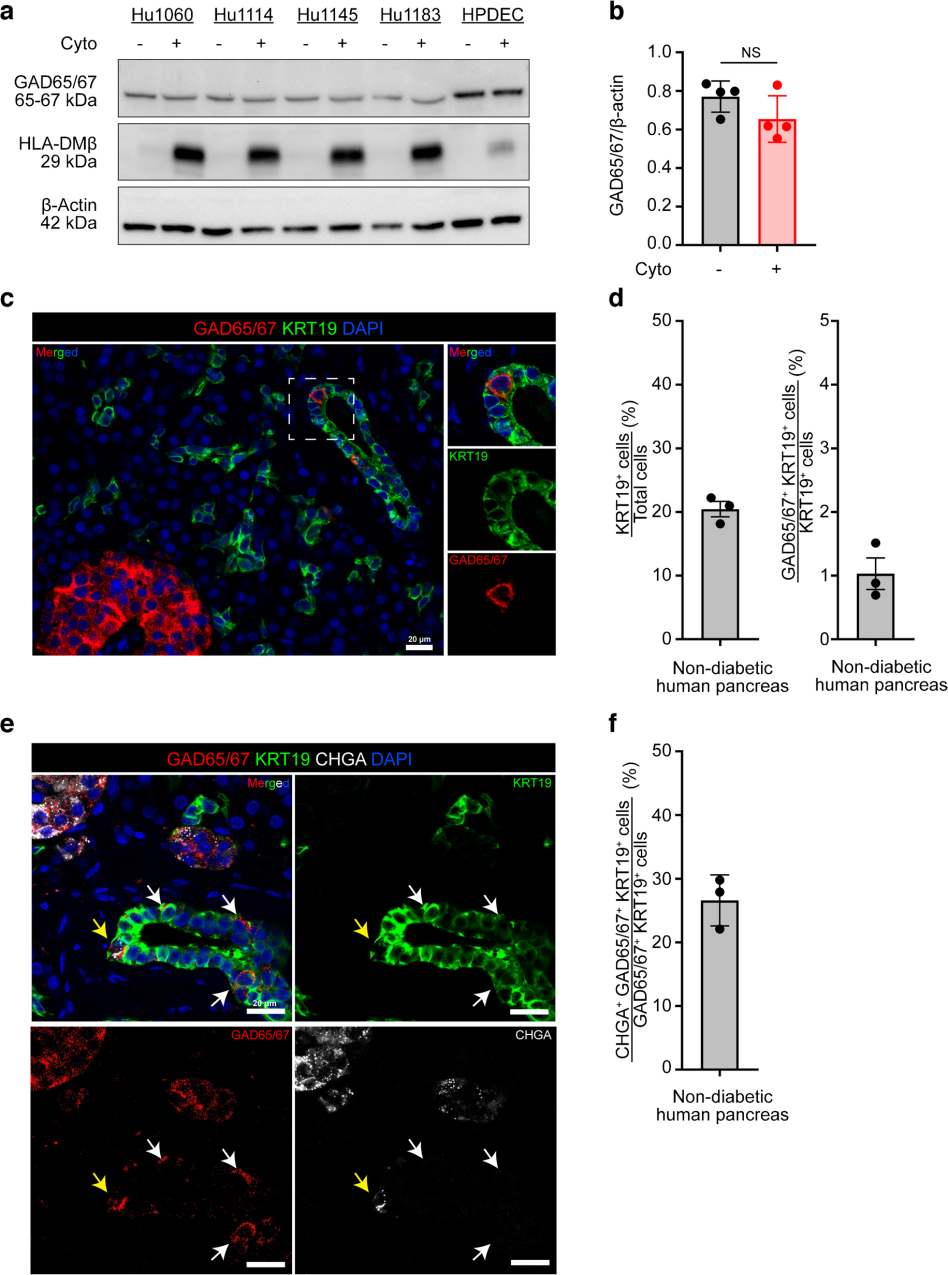
Ductal cells of non-diabetic donors express GAD65/67 protein. (**a**) Western blot analysis of the expression of GAD65/67, HLA-DMβ and β-actin in human ductal cells from suspension culture treated with vehicle or cytokines for 48 h (*N*=4 donors). HPDEC is an immortalised human ductal cell line. (**b**) Densitometry of GAD65/67 protein expression in human ductal cells. Densitometry values were normalised to β-actin housekeeping protein. *N*=4 donors. Data represent mean ± SD. Unpaired *t* test was used for statistical analysis. NS, not significant (*p*>0.05). (**c**) Formalin-fixed, paraffin-embedded pancreas tissue sections from donors without apparent diseases were co-stained for KRT19 (green), GAD65/67 (red) and nuclei (blue; stained with DAPI). A representative image is shown. (**d**) Quantification of ductal cells (KRT19^+^DAPI^+^) among total pancreatic cells (DAPI^+^) or GAD65/67^+^ ductal cells (GAD65/67^+^KRT19^+^DAPI^+^) among total ductal cells. *N*=3 donors. Each dot represents a donor, with value as the mean of *n*=2–3 technical replicates. Data represent mean ± SEM. (**e**) From the same donors as (**c**) and (**d**), pancreas tissue sections were co-stained for KRT19 (green), GAD65/67 (red), CHGA (white) and nuclei (blue; stained with DAPI). A representative image is shown. (**f**) Quantification of CHGA^+^ GAD65/67^+^ ductal cells (CHGA^+^GAD65/67^+^KRT19^+^DAPI^+^) among total GAD65/67^+^ ductal cells. *N*=3 donors. Each dot represents a donor, with value derived from *n*=1–2 technical replicates. Data represent mean ± SD. Scale bars, 20 μm. DAPI, 4′,6-diamidino-2-phenylindole

To assess GAD protein in ductal cells, we performed western blot analysis using the C-9 monoclonal antibody that detects both GAD65 and GAD67 (*N*=4 donors) (Fig. 5a, b); cytokine treatment did not alter GAD levels (Fig. 5a, b, ESM Fig. 9b). In contrast, HLA-DMβ was increased by cytokines (Fig. 5a, b, ESM Fig. 9c, d), as expected (Fig. 1i).

Co-immunofluorescence staining on human pancreas slides from three donors without apparent diseases revealed ~0.9% of KRT19^+^ ductal cells expressing GAD65/67 protein (Fig. 5c, d), while secondary-antibody-only controls showed minimal background staining (ESM Fig. 9e). As expected, islet cells also stained positive for GAD65/67 (Fig. 5c). This is consistent with our scRNA-seq data showing that ~1% of total ductal cells expressed *GAD1* transcripts (ESM Fig. 9a). Interestingly, 26.6% of KRT19^+^ GAD65/67^+^ ductal cells expressed CHGA, an endocrine marker (Fig. 5e, f), suggesting potential ductal-to-endocrine transdifferentiation [30]. Secondary-antibody-only controls showed minimal background staining (ESM Fig. 9f). Taken together, these findings demonstrate a subset of human ductal cells express *GAD1* mRNA and GAD65/67 protein.

## Discussion

In this study, we demonstrate that primary human ductal cells from non-diabetic donors respond to type 1 diabetes-associated proinflammatory cytokines in two key aspects. First, cytokines upregulate molecules involved in antigen presentation (HLA-DR and HLA-DQ), antigen processing (cathepsin S, HLA-DMα and HLA-DMβ) and co-stimulation (CD40 and ICAM-1) at both gene and protein levels (Figs 1, 2). scRNA-seq analysis from 86 donors confirmed a strong positive association of cytokine response and APC gene scores in individual ductal cells (Fig. 1). CD40 and ICAM-1 support sustained T cell responses [40, 41], preferentially CD4^+^ T cells [42], whereas CD80 and CD86 are central to naive T cell activation [40, 43]. Consistent with this, cytokine-treated ductal cells induce proliferation of the three CD4^+^ T cell clones tested (Figs 3, 4, ESM Figs 6–8), presumably through CD40 and ICAM-1 upregulation (Fig. 2c,d). In contrast, CD80 and CD86 were minimally expressed in ductal cells and did not respond to cytokines (ESM Fig. 3e,f). These results suggest that differential co-stimulatory molecule expression by ductal cells may impact downstream immune activation, which warrants further investigation.

Second, cytokine-treated ductal cells present exogenous GAD65 and HA peptides to GAD65- and HA-specific CD4^+^ T cell clones, respectively, inducing activation and proliferation (Figs 3, 4, ESM Fig. 7). These ductal cells were comparable to, and in some instances more effective than, the BSM APC cell line (ESM Fig. 6). Our findings establish cytokine-stimulated human pancreatic ductal cells as non-professional APCs.

Non-professional APCs have been described in many tissues. For example, intestinal, colonic and lung epithelial cells upregulate HLA class II under inflammatory stress [9, 10, 44], while lymph node stromal cells [45] and endothelial cells [46] can present antigens to T cells. Functionally, primary colonic epithelial cells from individuals with inflammatory bowel disease stimulate proliferation of CD4^+^ T cells from autologous or allogeneic peripheral blood [10]. Our study parallels these findings and adds pancreatic ductal epithelial cells to this expanding list of non-professional APCs.

Specific *HLA-DR* and *HLA-DQ* alleles*,* particularly HLA-DR4-DQ8 and HLA-DR3-DQ2 haplotypes, have strong association with type 1 diabetes [1, 2, 7]. We found that human pancreatic ductal cells upregulate HLA-DR and HLA-DQ transcripts and proteins in response to type 1 diabetes-associated cytokines (Figs 1, 2). While recent scRNA-seq studies [47, 48] reported HLA class II and antigen processing gene expression in cytokine-treated ductal cells, protein expression or functional antigen presentation was not confirmed. Here we show that cytokine-stimulated ductal cells express both the genes and proteins required for antigen presentation, processing and loading (Figs 1, 2), and can present an exogenous GAD65 peptide to CD4^+^ T cells (Figs 3, 4). Notably, Maestas et al [47] used TNF-α, IL-1β and IFN-γ at 150-, 500- and 80-fold higher concentrations, respectively, than in this study, suggesting the sensitivity of primary ductal cells. Experimentally, destruction of rat beta cells can release GAD into culture medium [49]. This suggests that ductal cells encountering extracellular GAD from neighbouring beta cells in a type 1 diabetes microenvironment may present antigen via HLA-DR/DQ, potentially amplifying autoimmunity.

Unexpectedly, ductal cells may also process and present endogenous GAD65/67 protein (Figs 3, 4). Approximately 0.9% of KRT19^+^ ductal cells from donors without apparent diseases stained positive for GAD65/67 protein (Fig. 5). Given the cytokine-induced upregulation of antigen-processing molecules in ductal cells (Figs 1, 2), it is plausible that cytokine-exposed ductal cells process and present endogenously derived GAD65/67 peptides to CD4^+^ T cells. However, further investigation is required.

Several limitations should be acknowledged. First, this study examined only GAD65 autoantigen; whether ductal cells present additional type 1 diabetes-relevant autoantigens remains unknown. Second, in vitro coculture models may not fully recapitulate in vivo microenvironment. Third, using dissociated human ductal cells precludes assessment of spatial relationships with lymphoid and islet cells. Notably, extra-islet lymphoid structures have recently been identified in type 1 diabetes pancreas [50]. Despite these limitations, to the best of our knowledge, this study provides the first evidence that pancreatic ductal cells can present antigens in response to type 1 diabetes-associated inflammatory cytokines. Additionally, our 3D suspension culture platform of primary human ductal cells may help reduce animal use in research.

## Supplementary Information

Below is the link to the electronic supplementary material.

Supplementary file1 (PDF 6.82 MB)

Supplementary file2 (XLSX 353 KB)

## Data Availability

Further information and requests for resources and reagents should be directed to and will be fulfilled by the Lead Contact, HTK (hku@coh.org). The bulk mRNA-seq dataset is deposited to the Gene Expression Omnibus (GEO; https://www.ncbi.nlm.nih.gov/geo/) with accession number GSE309294. The PanKbase Seurat object for publicly available scRNA-seq datasets [11, 20, 22] is archived at https://pubrepo.coh.org/publication/17 and code to reproduce the associated figures is available at https://github.com/KaddisLab/2026_erdem_et_al_diabetologia.

## Abbreviations

AABP: Autoantibody-positive individuals
APC: Antigen-presenting cell
B-LCL: B lymphoblastoid cell line
CFSE: Carboxyfluorescein succinimidyl ester
DEG: Differentially expressed gene
FC: Fold change
GAD65: Glutamic acid decarboxylase 65 kDa isoform
GAD67: Glutamic acid decarboxylase 67 kDa isoform
GSEA: Gene set enrichment analysis
HA: Haemagglutinin
HLA: Human leukocyte antigen
HPDEC: Human pancreatic ductal epithelial cell
MFI: Mean fluorescence intensity
NODM: Individuals with no diabetes mellitus
qRT-PCR: Quantitative PCR with reverse transcription
scRNA-seq: Single-cell RNA-seq
*t*-SNE: *t*-Distributed stochastic neighbour embedding

## References

[1] James EA, Joglekar AV, Linnemann AK, Russ HA, Kent SC (2023) The beta cell-immune cell interface in type 1 diabetes (T1D). Mol Metab 78:101809. 10.1016/j.molmet.2023.101809 37734713 10.1016/j.molmet.2023.101809 PMC10622886

[2] Pugliese A (2017) Autoreactive T cells in type 1 diabetes. J Clin Investig 127(8):2881–2891. 10.1172/JCI94549 28762987 10.1172/JCI94549 PMC5531393

[3] Creusot RJ, Postigo-Fernandez J, Teteloshvili N (2018) Altered function of antigen-presenting cells in type 1 diabetes: a challenge for antigen-specific immunotherapy? Diabetes 67(8):1481–1494. 10.2337/db17-1564 30030289 10.2337/db17-1564 PMC6054431

[4] Kent SC, Mannering SI, Michels AW, Babon JAB (2017) Deciphering the pathogenesis of human type 1 diabetes (T1D) by interrogating T cells from the “scene of the crime.” Curr Diabetes Rep 17(10):95. 10.1007/s11892-017-0915-y 10.1007/s11892-017-0915-y PMC5600889 28864875

[5] Reijonen H, Mallone R, Heninger AK et al (2004) GAD65-specific CD4^+^ T-cells with high antigen avidity are prevalent in peripheral blood of patients with type 1 diabetes. Diabetes 53(8):1987–1994. 10.2337/diabetes.53.8.1987 15277377 10.2337/diabetes.53.8.1987

[6] Nanaware PP, Calvo-Calle JM, Redick SD et al (2025) The antigen presentation landscape of cytokine-stressed human pancreatic islets. Cell Rep 44(8):115927. 10.1016/j.celrep.2025.115927 40684438 10.1016/j.celrep.2025.115927 PMC12624573

[7] Ilonen J, Kiviniemi M, Lempainen J et al (2016) Genetic susceptibility to type 1 diabetes in childhood - estimation of HLA class II associated disease risk and class II effect in various phases of islet autoimmunity. Pediatric Diabetes 17(Suppl 22):8–16. 10.1111/pedi.12327 27411431 10.1111/pedi.12327

[8] Rodriguez-Calvo T, Ekwall O, Amirian N, Zapardiel-Gonzalo J, von Herrath MG (2014) Increased immune cell infiltration of the exocrine pancreas: a possible contribution to the pathogenesis of type 1 diabetes. Diabetes 63(11):3880–3890. 10.2337/db14-0549 24947367 10.2337/db14-0549 PMC4207385

[9] Beyaz S, Chung C, Mou H et al (2021) Dietary suppression of MHC class II expression in intestinal epithelial cells enhances intestinal tumorigenesis. Cell Stem Cell 28(11):1922-1935 e1925. 10.1016/j.stem.2021.08.007 34529935 10.1016/j.stem.2021.08.007 PMC8650761

[10] Dotan I, Allez M, Nakazawa A, Brimnes J, Schulder-Katz M, Mayer L (2007) Intestinal epithelial cells from inflammatory bowel disease patients preferentially stimulate CD4^+^ T cells to proliferate and secrete interferon-γ. Am J Physiol Gastrointest Liver Physiol 292(6):G1630-1640. 10.1152/ajpgi.00294.2006 17347451 10.1152/ajpgi.00294.2006

[11] Fasolino M, Schwartz GW, Patil AR et al (2022) Single-cell multi-omics analysis of human pancreatic islets reveals novel cellular states in type 1 diabetes. Nat Metab 4(2):284–299. 10.1038/s42255-022-00531-x 35228745 10.1038/s42255-022-00531-x PMC8938904

[12] Pujol-Borrell R, Todd I, Doshi M, Gray D, Feldmann M, Bottazzo GF (1986) Differential expression and regulation of MHC products in the endocrine and exocrine cells of the human pancreas. Clin Exp Immunol 65(1):128–139 3098471 PMC1542269

[13] Jalleh RP, Gilbertson JA, Williamson RC, Slater SD, Foster CS (1993) Expression of major histocompatibility antigens in human chronic pancreatitis. Gut 34(10):1452–1457. 10.1136/gut.34.10.1452 8244120 10.1136/gut.34.10.1452 PMC1374561

[14] Bedossa P, Bacci J, Lemaigre G, Martin E (1990) Lymphocyte subsets and HLA-DR expression in normal pancreas and chronic pancreatitis. Pancreas 5(4):415–420. 10.1097/00006676-199007000-00007 1974351 10.1097/00006676-199007000-00007

[15] Eizirik DL, Colli ML, Ortis F (2009) The role of inflammation in insulitis and β-cell loss in type 1 diabetes. Nat Rev Endocrinol 5(4):219–226. 10.1038/nrendo.2009.21 19352320 10.1038/nrendo.2009.21

[16] Qi M, Valiente L, McFadden B et al (2015) The choice of enzyme for human pancreas digestion is a critical factor for increasing the success of islet isolation. Transplant Direct 1(4):e14. 10.1097/TXD.0000000000000522 26146662 10.1097/TXD.0000000000000522 PMC4486320

[17] Zook HN, Quijano JC, Ortiz JA et al (2024) Activation of ductal progenitor-like cells from adult human pancreas requires extracellular matrix protein signaling. iScience 27(3):109237. 10.1016/j.isci.2024.109237 38433896 10.1016/j.isci.2024.109237 PMC10904999

[18] Zook HN, Quijano JC, Ortiz JA, Donohue C, Erdem N, Ku HT (2025) Protocol to study ductal progenitor-like cells from the adult human pancreas using 3D suspension and methylcellulose-based culture systems. STAR Protoc 6(2):103847. 10.1016/j.xpro.2025.103847 40424137 10.1016/j.xpro.2025.103847 PMC12152497

[19] Ortiz JA, Ghazalli N, Lopez K et al (2024) Trefoil factor 2 expressed by the murine pancreatic acinar cells is required for the development of islets and for β-cell function during aging. Diabetes 73(9):1447–1461. 10.2337/db23-0490 38905124 10.2337/db23-0490 PMC11333379

[20] Vu HTH, Sun HAN, Sharp S et al (2025) 2121-LB: PanKbase integrated single-cell map—a comprehensive atlas of human pancreatic islets unlocking insights into type 1 and type 2 diabetes. Diabetes 74(Suppl 1):2121-LB. 10.2337/db25-2121-LB

[21] Kaestner KH, Powers AC, Naji A, Atkinson MA (2019) NIH Initiative to improve understanding of the pancreas, islet, and autoimmunity in type 1 diabetes: the Human Pancreas Analysis Program (HPAP). Diabetes 68(7):1394–1402. 10.2337/db19-0058 31127054 10.2337/db19-0058 PMC6609987

[22] Patil AR, Schug J, Naji A, Kaestner KH, Faryabi RB, Vahedi G (2023) Single-cell expression profiling of islets generated by the Human Pancreas Analysis Program. Nat Metab 5(5):713–715. 10.1038/s42255-023-00806-x 37188822 10.1038/s42255-023-00806-x PMC10731597

[23] Schneider CA, Rasband WS, Eliceiri KW (2012) NIH Image to ImageJ: 25 years of image analysis. Nat Methods 9(7):671–675. 10.1038/nmeth.2089 22930834 10.1038/nmeth.2089 PMC5554542

[24] Mellado-Gil J, Rosa TC, Demirci C et al (2011) Disruption of hepatocyte growth factor/c-Met signaling enhances pancreatic β-cell death and accelerates the onset of diabetes. Diabetes 60(2):525–536. 10.2337/db09-1305 20980460 10.2337/db09-1305 PMC3028352

[25] Kondegowda NG, Filipowska J, Do JS et al (2023) RANKL/RANK is required for cytokine-induced beta cell death; osteoprotegerin, a RANKL inhibitor, reverses rodent type 1 diabetes. Sci Adv 9(44):eadf5238. 10.1126/sciadv.adf5238 37910614 10.1126/sciadv.adf5238 PMC10619938

[26] Chang CH, Fontes JD, Peterlin M, Flavell RA (1994) Class II transactivator (CIITA) is sufficient for the inducible expression of major histocompatibility complex class II genes. J Exp Med 180(4):1367–1374. 10.1084/jem.180.4.1367 7931070 10.1084/jem.180.4.1367 PMC2191681

[27] Pishesha N, Harmand TJ, Ploegh HL (2022) A guide to antigen processing and presentation. Nat Rev Immunol 22(12):751–764. 10.1038/s41577-022-00707-2 35418563 10.1038/s41577-022-00707-2

[28] Chen L, Flies DB (2013) Molecular mechanisms of T cell co-stimulation and co-inhibition. Nat Rev Immunol 13(4):227–242. 10.1038/nri3405 23470321 10.1038/nri3405 PMC3786574

[29] Lee J, Sugiyama T, Liu Y et al (2013) Expansion and conversion of human pancreatic ductal cells into insulin-secreting endocrine cells. eLife 2:e00940. 10.7554/eLife.00940 24252877 10.7554/eLife.00940 PMC3826580

[30] Quijano JC, Wedeken L, Ortiz JA et al (2023) Methylcellulose colony assay and single-cell micro-manipulation reveal progenitor-like cells in adult human pancreatic ducts. Stem Cell Rep 18(3):618–635. 10.1016/j.stemcr.2023.02.001 10.1016/j.stemcr.2023.02.001 PMC10031308 36868230

[31] Furukawa T, Duguid WP, Rosenberg L, Viallet J, Galloway DA, Tsao MS (1996) Long-term culture and immortalization of epithelial cells from normal adult human pancreatic ducts transfected by the E6E7 gene of human papilloma virus 16. Am J Pathol 148(6):1763–1770 8669463 PMC1861644

[32] Atkinson MA, Kaufman DL, Campbell L et al (1992) Response of peripheral-blood mononuclear cells to glutamate decarboxylase in insulin-dependent diabetes. Lancet 339(8791):458–459. 10.1016/0140-6736(92)91061-c 1346821 10.1016/0140-6736(92)91061-c

[33] Oling V, Marttila J, Ilonen J et al (2005) GAD65- and proinsulin-specific CD4^+^ T-cells detected by MHC class II tetramers in peripheral blood of type 1 diabetes patients and at-risk subjects. J Autoimmun 25(3):235–243. 10.1016/j.jaut.2005.09.018 16263242 10.1016/j.jaut.2005.09.018

[34] Turner TR, Hayhurst JD, Hayward DR et al (2018) Single molecule real-time DNA sequencing of HLA genes at ultra-high resolution from 126 International HLA and Immunogenetics Workshop cell lines. HLA 91(2):88–101. 10.1111/tan.13184 29171935 10.1111/tan.13184

[35] Roche PA, Furuta K (2015) The ins and outs of MHC class II-mediated antigen processing and presentation. Nat Rev Immunol 15(4):203–216. 10.1038/nri3818 25720354 10.1038/nri3818 PMC6314495

[36] Novak EJ, Liu AW, Nepom GT, Kwok WW (1999) MHC class II tetramers identify peptide-specific human CD4^+^ T cells proliferating in response to influenza A antigen. J Clin Investig 104(12):R63-67. 10.1172/JCI8476 10606632 10.1172/JCI8476 PMC480919

[37] Cram DS, Faulkner-Jones B, Kun J, Harrison LC (1995) Glutamic acid decarboxylase-67 (GAD67): expression relative to GAD65 in human islets and mapping of autoantibody epitopes. Endocrinology 136(3):1111–1119. 10.1210/endo.136.3.7532577 7532577 10.1210/endo.136.3.7532577

[38] Segerstolpe A, Palasantza A, Eliasson P et al (2016) Single-cell transcriptome profiling of human pancreatic islets in health and type 2 diabetes. Cell Metab 24(4):593–607. 10.1016/j.cmet.2016.08.020 27667667 10.1016/j.cmet.2016.08.020 PMC5069352

[39] Soghomonian JJ, Martin DL (1998) Two isoforms of glutamate decarboxylase: why? Trends Pharmacol Sci 19(12):500–505. 10.1016/s0165-6147(98)01270-x 9871412 10.1016/s0165-6147(98)01270-x

[40] Howland KC, Ausubel LJ, London CA, Abbas AK (2000) The roles of CD28 and CD40 ligand in T cell activation and tolerance. J Immunol 164(9):4465–4470. 10.4049/jimmunol.164.9.4465 10779746 10.4049/jimmunol.164.9.4465

[41] Chirathaworn C, Kohlmeier JE, Tibbetts SA, Rumsey LM, Chan MA, Benedict SH (2002) Stimulation through intercellular adhesion molecule-1 provides a second signal for t cell activation. J Immunol 168(11):5530–5537. 10.4049/jimmunol.168.11.5530 12023348 10.4049/jimmunol.168.11.5530

[42] Cayabyab M, Phillips JH, Lanier LL (1994) CD40 preferentially costimulates activation of CD4^+^ T lymphocytes. J Immunol 152(4):1523–1531 7509825

[43] Bour-Jordan H, Bluestone JA (2002) CD28 function: a balance of costimulatory and regulatory signals. J Clin Immunol 22(1):1–7. 10.1023/A:1014256417651 11958588 10.1023/a:1014256417651

[44] Debbabi H, Ghosh S, Kamath AB et al (2005) Primary type II alveolar epithelial cells present microbial antigens to antigen-specific CD4^+^ T cells. Am J Physiol Lung Cell Mol Physiol 289(2):L274-279. 10.1152/ajplung.00004.2005 15833765 10.1152/ajplung.00004.2005

[45] Hirosue S, Dubrot J (2015) Modes of antigen presentation by lymph node stromal cells and their immunological implications. Front Immunol 6:446. 10.3389/fimmu.2015.00446 26441957 10.3389/fimmu.2015.00446 PMC4561840

[46] Knolle PA, Schmitt E, Jin S et al (1999) Induction of cytokine production in naive CD4^+^ T cells by antigen-presenting murine liver sinusoidal endothelial cells but failure to induce differentiation toward Th1 cells. Gastroenterology 116(6):1428–1440. 10.1016/s0016-5085(99)70508-1 10348827 10.1016/s0016-5085(99)70508-1

[47] Maestas MM, Ishahak M, Augsornworawat P et al (2024) Identification of unique cell type responses in pancreatic islets to stress. Nat Commun 15(1):5567. 10.1038/s41467-024-49724-w 38956087 10.1038/s41467-024-49724-w PMC11220140

[48] Munoz Garcia A, Juksar J, Groen N, Zaldumbide A, de Koning E, Carlotti F (2024) Single-cell transcriptomics reveals a role for pancreatic duct cells as potential mediators of inflammation in diabetes mellitus. Front Immunol 15:1381319. 10.3389/fimmu.2024.1381319 38742118 10.3389/fimmu.2024.1381319 PMC11089191

[49] Smismans A, Ling Z, Pipeleers D (1996) Damaged rat β cells discharge glutamate decarboxylase in the extracellular medium. Biochem Biophys Res Commun 228(2):293–297. 10.1006/bbrc.1996.1655 8920908 10.1006/bbrc.1996.1655

[50] Korpos E, Kadri N, Loismann S et al (2021) Identification and characterisation of tertiary lymphoid organs in human type 1 diabetes. Diabetologia 64(7):1626–1641. 10.1007/s00125-021-05453-z 33912981 10.1007/s00125-021-05453-z PMC8187221

