# Supplementary material for: Human pancreatic ductal cells from non-diabetic donors function as non-professional antigen-presenting cells upon inflammatory cytokine exposure
Source: Diabetologia. 2026 May 7;69(8):2323–37. doi: 10.1007/s00125-026-06746-x (PMC13310231; doi:10.1007/s00125-026-06746-x)
Supplement: Supplementary file 1 — Supplementary file1 (PDF 6.82 MB) [file 125_2026_6746_MOESM1_ESM.pdf]

Supplemental Information

Human pancreatic ductal cells from non-diabetic donors function as non-professional antigen presenting cells upon inflammatory cytokine exposure

Neslihan Erdem, David Arribas-Layton, Heather N. Zook, Denis O'Meally, Jacob Mares, Janine C. Quijano, Cecile Donohue, Jose A. Ortiz, Kevin Jou, Rupangi C. Vasavada, Enrique Montero, John S. Kaddis, Helena Reijonen, Hsun Teresa Ku

This document contains the following information:

Supplemental Materials and Methods Pages 2 to 14

Supplemental Figures and Legends Pages 15 to 32

References Pages 32 to 35

Supplemental Materials and Methods

Primary human pancreatic exocrine cells.

Procurement

Human pancreas from deceased organ donors without diabetes were collected and shipped to the Southern California Islet Cell Resource Center (SC-ICRC) at the City of Hope, where islet isolation was carried out following previously established protocols [1]. After islet extraction, de-identified pancreatic exocrine tissues **(ESM Human Tissue Checklist, ESM Table 1 and 2)** were provided to HTK.'s laboratory under an IRB Protocol #23728, classified as Non-Human Subjects Research.

Dissociation and cryopreservation

The procedures for dissociating and cryopreserving exocrine tissues have been described in detail in our prior publications [2-4]. Briefly, pancreatic exocrine tissue was rinsed in cold phosphate-buffered saline (PBS) and then resuspended in Dulbecco's PBS (Corning, cat# 21-031-CV) containing 0.1% bovine serum albumin (BSA) (Sigma-Aldrich, cat# A8412), 2-4 mg/ml collagenase B (Sigma-Aldrich, cat# 11088831001) and 2000 IU/ml DNase I (Millipore Sigma, cat# 260913). Tissues were incubated at 37°C in a water bath for 10 min, followed by gentle disruption using a 16½ G syringe needle. Single-cell suspension was obtained by filtering through 100 µm followed by 40 µm nylon meshes. Dissociated cells were counted, gently resuspended in Cryostor CS10 (Biolife Solutions, cat# 210102), and frozen using a CryoMed Controlled-Rate Freezer (Thermo Scientific, Waltham, USA). Once frozen, the vials were transferred to a liquid nitrogen tank for long-term storage.

3D suspension culture of primary human ductal cells.

Cryovials containing dissociated exocrine cells were thawed and cultured according to our prior publications [2, 3]. Briefly, frozen vials were placed in a 37°C water bath for ~2 min or until a small chuck of ice remained visible. Cells were washed once with warm PBS supplemented with 0.1% BSA. The cell pellets were gently resuspended in pre-warmed suspension culture medium composed of DMEM/F12, 10% KnockOut Serum Replacement, Penicillin-Streptomycin (PS), nicotinamide, Noggin, epidermal growth factor (EGF), A83-01, R-spondin1 (RSPO1), gastrin II sulfated, and Y-27632 (**ESM Table 3**). Cells were plated in ultra-low binding plates and incubated at 37°C with 5% CO_2.

During the initial overnight incubation (up to 16 h), Y-27632 was added to the culture medium. On day 1, cells were collected and washed with warm DMEM/F12 containing 0.1% BSA and PS. Subsequently, live cells were enriched using Histopaque 1077 density gradient medium (Sigma-Aldrich, cat# 10771-100ML). After enrichment, cells were resuspended in fresh suspension medium without Y-27632. Experiments in this study were not masked, and the samples were not randomized. Only tissues from donors without diabetes were studied.

Primary human CD4$^+$ T cell clones.

The isolation of the CD4$^+$ T cell clones BRI-4.13 (GAD65-specific and HLA-DRB1*04:01-restricted) and BRI-5.325 (GAD65-specific and HLA-DRB1*04:04-restricted) have been described previously [5-7]. Briefly, monomers composed of biotinylated MHC class II molecules with HLA-DRB1*04:01 or HLA-DRB1*04:04 specificity were produced (Benaroya Research Institute, Seattle, Washington) and loaded with a peptide sequence corresponding to amino acid residues 555-567 (557I) (NFIRMVISNPAAT) of glutamic acid decarboxylase 65 kD isoform (GAD65). Peptide-loaded monomers were then assembled into tetramers by binding with fluorochrome-conjugated streptavidin. Peripheral blood mononuclear cells (PBMCs) of a T1D donor with the HLA-DRB1*04:01 or HLA-DRB1*04:04 haplotype were isolated and cultured with GAD65 557I peptide for approximately two weeks. Subsequently, cells were stained with

tetramers and anti-CD4 antibody and analyzed by flow cytometry. Tetramer$^+$ CD4$^+$ T cells were single cell sorted into a 96-well plate, expanded, and cryopreserved. The specificity of the clones has been confirmed by wild-type peptide-specific proliferation, cytokine production and tetramer staining as previously described [7].

For Hemaglutinin (HA)-specific CD4$^+$ T cell clone (HLA-DRB1*04:04-restricted), monomers composed of biotinylated MHC class II molecules with HLA-DRB1*04:04 specificity were produced (Benaroya Research Institute, Seattle, Washington) and loaded with a peptide sequence corresponding to amino acid residues HA306 (PKYVKQNTLKLAT) of HA. Peptide-loaded monomers were then assembled into tetramers by binding with fluorochrome-conjugated streptavidin. Peripheral blood mononuclear cells (PBMCs) of a healthy donor with the HLA-DRB1*04:04, HLA-DRB1*03:01 haplotype were isolated and cultured with HA306 peptide for approximately two weeks. Subsequently, cells were stained with tetramers and anti-CD4 antibody and analyzed by flow cytometry. Tetramer$^+$ CD4$^+$ T cells were single cell sorted into a 96-well plate expanded, and cryopreserved. The specificity of the clones has been confirmed by tetramer staining.

Cell lines.

The HPDEC cell line, an immortalized human pancreatic ductal epithelial cell line, was obtained from AddexBio (cat# T0018001) and cultured according to manufacturer's recommendations. Mycoplasma negativity was validated by the manufacturer. Briefly, cryopreserved HPDEC cells were thawed and gently resuspended in Keratinocyte Serum-Free Medium (KSFM, Gibco, cat# 17005042) supplemented with 50 µg/ml bovine pituitary extract, 5 ng/ml epidermal growth factor (EGF), and 1% Penicillin-Streptomycin (Gibco, cat# 15140122). Cells were seeded at 1 x 10^6 cells in 15 ml KSFM into a T75 tissue culture (TC) treated flask and incubated at 37°C with 5% CO_2. Medium was replaced every 2-3 days, and cells were passaged

upon reaching approximately 80% confluency. For this study, cells were received from the manufacturer at passage 5, and cells between passages 6-10 were used in the experiments.

The BSM cell line, an Epstein-Barr virus (EBV) transformed B lymphoblastoid cell line (B-LCL), was obtained from Sigma (cat# 88052032). BSM cells are homozygous for HLA-DRB1*04:01. Mycoplasma negativity was confirmed using MycoAlert Mycoplasma Detection Kit (Lonza, cat #LT07-318), and HLA typing confirmed the haplotype. Cryopreserved BSM cells were thawed and gently resuspended in RPMI 1640 (Gibco, cat# 11875093) supplemented with 10% human heat inactivated serum (Millipore Sigma, cat# H3667), 1% Penicillin-Streptomycin (Gibco, cat# 15140122), and 1% L-Glutamine (Gibco, cat# A2916801). Cells were cultured at 37°C with 5% CO_2 [8].

Proinflammatory cytokine treatment.

After live cell enrichment using Histopaque 1077, day 1 ductal cells were resuspended in proinflammatory cytokines, TNF-α, IL-1β, and IFN-γ, at doses indicated in **ESM Table 4**, and cultured for 48 h. HPDEC cell line was treated with the 250× cytokine dose for 48 h.

Quantitative PCR with reverse transcription (qRT-PCR).

qRT-PCR was performed as previously described [9]. Briefly, equal amounts of RNA were used for reverse transcription to complementary (c)DNA using the QuantiTect Reverse Transcription Kit (Qiagen, cat# 205313). The resulting cDNA was used in PCR reactions and water was used as negative control. PCR reactions were set up in a MicroAmp Optical 384-well plate, and thermal cycling was carried out on the Applied Biosystems ViiA 7 system as follows: initial denaturation at 95°C for 2 minutes, followed by 45 cycles of 94°C for 30 seconds, 60°C for 1 minute, and 72°C for 10 seconds, with a final extension at 72°C for 2 minutes. Gene expressions were assessed using predesigned TaqMan probes (Thermo Fisher Scientific, Waltham, USA) as

listed in **ESM Table 5**. The housekeeping gene *ACTB* served as an internal reference for normalization. All PCR runs included two or three technical replicates.

Bulk RNA-sequencing.

RNA quality control (QC) was performed using the Agilent Tapestation RNA Tape. Samples with RNA integrity number (RIN) > 7 were selected for sequencing. For each sample, a total of 250 ng of RNA was used to prepare sequencing library using the KAPA mRNA HyperPrep Kit (Roche, cat# 08098123702), following the manufacturer's protocol. Final libraries were validated using the Agilent Bioanalyzer DNA High Sensitivity Kit and quantified with Qubit. Sequencing was conducted on the NovaSeq X Series platform using the 25B reagent kit (300 cycles). NovaSeq Control Software 1.2.2.48004 was used for sequencing, and Illumina BCL Convert v4.2.7 was employed to convert base call (BCL) files into FASTQ format.

RNA-Seq reads were trimmed to remove sequencing adapters using Trimmomatic [10] and polyA tails using FASTP [11]. The processed reads were mapped back to the human genome (hg38) using STAR software (v. 2.6.0.a) [12]. The HTSeq software (v.0.11.1) [13] was applied to generate the count matrix, with default parameters.

Differential expression analysis was performed by normalizing the raw read counts to expression values using the trimmed mean of M-values (TMM) normalization method implemented in edgeR [14]. Specifically, for paired comparisons between experimental conditions, generalized linear models were employed to identify differentially expressed genes (DEGs), with TMM-normalized expression levels serving as the dependent variable and experimental condition as the independent variable. Pairing factors (e.g., individual donors) were included in the model to minimize confounding effects. A ranked list of genes according to their log_2FC and p-values are provided in **ESM Tables 6-7**.

Pathway analysis was conducted using the GSEA algorithm implemented in clusterProfiler (v.4.14.3) package in R [15-18]. Significantly enriched pathways are presented in **ESM Tables 8-19**.

Single-cell RNA-sequencing analysis of ductal cells.

Data Source and Sample Selection

Single-cell RNA sequencing data from human pancreatic islets were obtained from the Human Pancreas Analysis Program (HPAP) and published studies via the PanKbase repository [19-24]. The following exclusion criteria were applied: (1) type 2 diabetes donors; (2) islet samples from donors that were subject to ex-vivo treatments; and (3) high-risk individuals without diabetes (BMI ≥ 35 and HbA_{1c} ≥ 6.0%), who may have undiagnosed metabolic dysfunction.

The final analysis included 86 donors (20,605 ductal cells), comprising individuals without diabetes (NODM), autoantibody-positive (AABP), and with type 1 diabetes (T1DM). Donor characteristics are provided in **ESM Table 20**.

Gene Signature Scoring

Cytokine response score was quantified using a 6-gene signature (*CXCL9, CXCL10, CXCL11, GBP4, GBP5, NOS2*) derived from bulk RNA-seq analysis of cytokine-treated human ductal cells (**ESM Table 6**). Antigen presenting cell (APC) score was quantified using an 18-gene signature encompassing HLA class II (*HLA-DRA, HLA-DRB1, HLA-DRB5, HLA-DQA1, HLA-DQA2, HLA-DQB1, HLA-DPA1, HLA-DPB1, CIITA*), co-stimulatory (*CD40, ICAM1*), and antigen processing/loading machinery (*CD74, CTSS, HLA-DMA, HLA-DMB, HLA-DOA, TAP1, TAP2*) (**ESM Table 6, Fig. 1g-i)**. Gene signature scores were calculated using UCell [25], which provides robust single-cell scoring based on the Mann-Whitney U statistic.

Statistical Analysis

To account for the large proportion of cells with zero APC scores and to address pseudoreplication arising from non-independence of cells within donors, the relationship between cytokine response and APC gene expression was assessed using a two-part mixed-effects hurdle model [26]. The model consists of two components:

Firstly, the binary component modelled the probability of APC program induction (i.e., the odds of a cell having an APC score > 0) as a function of cytokine exposure status. A mixed-effects logistic regression was fit to all 20,605 cells from 86 donors: P(APC > 0) ~ Cytokine Binary + (1 | Donor), where Cytokine Binary indicates whether a cell exhibited any detectable cytokine response (score > 0).

Secondly, the positive component modelled the magnitude of the APC response, conditional on the cell having both APC score and cytokine score greater than zero. Among cells with APC score >0 and cytokine score > 0 (108 cells from 33 donors), a linear mixed model tested whether cytokine magnitude predicts APC magnitude: APC Score ~ Cytokine Score + (1 | Donor). A sensitivity analysis applied inverse cell-count weights (1/n~cells per donor~) to equalize each donor's contribution regardless of the number of cells sampled (data not shown). Effect sizes were standardized (β × SDpredictor / SDoutcome) to enable comparison with correlation coefficients. The positive component of the hurdle model estimated a standardized effect size of β* = 0.45 [95% CI: 0.28–0.62], $p < 0.001$; **Fig. 1k**), indicating that a one SD increase in cytokine score corresponded to a 0.45-SD increase in APC score. Both components of the hurdle model included a random intercept for each donor to account for the hierarchical structure of the data (i.e., cells clustered within individuals). P-values for the fixed effects in both models were computed using Satterthwaite's degrees of freedom approximation, as implemented in the lmerTest R package [27]. The dose-response relationship from the positive component is visualized in scatter plots (**Fig. 1k**) using the APC score>0 and cytokine score>0 subset, with the standardized effect size (β*) and LMM fixed-effect trend line annotated directly on the figures.

Analysis of random slopes revealed significant heterogeneity in the cytokine-APC dose-response relationship across donors with APC score>0 (N=84 donors, likelihood ratio test $p<0.001$; **ESM Fig. 2a**), suggesting that additional donor-level factors modulate the strength of this association. Nevertheless, the main effect was positive for all donors.

To investigate potential sources of this heterogeneity, we tested for interaction effects between cytokine exposure and donor characteristics (age and diabetes status) in both hurdle model components. Neither showed significant effect modification (data not shown), indicating that the cytokine-APC relationship was consistent across donor age groups and disease states.

Software

All analyses were performed in R (version 4.4.1) using Seurat (v5.3.1) [26] for single-cell data handling, UCell (v2.10.1) [25] for signature scoring, glmmTMB (v1.1.10) [27] for the binary component of the hurdle model, lme4 (v1.1.35.5) [28] and lmerTest (v3.1.3) [29] for the positive component, and targets (v1.11.4) [30] for pipeline reproducibility.

Flow Cytometry.

For data in **Fig. 2** and **ESM Fig. 3**, cultured human ductal cells were washed with PBS and incubated with Zombie Aqua Dead Cell Stain (Biolegend, cat# 423101) diluted 1:1,000 in PBS for 15 min at RT to discriminate live and dead cells. Cells were washed with PBS and resuspended in flow cytometry buffer consisting of PBS with 2% fetal bovine serum (FBS). For surface marker detection, cells were incubated with fluorophore-conjugated or biotin-conjugated monoclonal antibodies **(ESM Table 21)** in 100 µl of flow cytometry buffer at 4°C for 30 min in the dark. During primary antibody staining, BD Horizon Brilliant Stain Buffer Plus (BD Biosciences, cat# 566385) was added to flow cytometry buffer according to manufacturer’s protocol to reduce staining artifacts due to the use of multiple BD Horizon Brilliant fluorescent polymer dyes. After 30 min on ice, cells were washed and incubated with streptavidin-labeled allophycocyanin (APC)

(Miltenyi, cat# 130-106-792) for 30 min at 4°C. After staining, cells were washed twice, centrifuged at 300 x g for 5 min, and resuspended in 200 μl of flow cytometry buffer. Cells were first gated on forward scatter (FSC) and side scatter (SSC) to exclude debris, followed by single and live (Zombie negative) cells **(ESM Fig. 3a)**. Baseline gating for each protein marker was established using fluorescence minus one (FMO) controls (**ESM Fig. 3b).**

For coculture experiments, cells collected at indicated time points were washed and stained with BD Via-Probe Cell Viability Solution (BD Biosciences, cat# 555815) and fluorochrome-conjugated antibodies for 20 min at 4°C in the dark. After staining, cells were washed twice, centrifuged at 300 x g for 5 min, and resuspended in 200 μl of flow cytometry buffer. For proliferation assay, $CD4^+$ T cells were stained with CellTrace CFSE (Thermo Fisher Scientific, cat# C34554) at 2.5 μM prior to coculture, and proliferation tracking was conducted according to manufacturer's protocol. The cell gating strategy is presented in **ESM Fig. 5a-c**. Antibodies used in flow cytometry analysis are listed in **ESM Table 21**.

Flow cytometry was performed using an Attune Nxt Acoustic Focusing Flow Cytometer System (Thermo Fisher Scientific, Waltham, USA). Data were analyzed using FlowJo v.10 (BD Biosciences, Franklin Lakes, USA). Mean fluorescence intensity (MFI) and percentage of positive cells were quantified according to the described gating strategies **(ESM Fig. 3a-b, 5a-c)**. For *t*-SNE analysis, the FlowJo v.10 *t*-SNE plugin was used to generate a two-dimensional representation of high-dimensional flow cytometry data using 1,000 iterations and a perplexity of 30. Cell populations defined by the manual gating strategy were projected onto the *t*-SNE map.

Western blot.

Cells were lysed using RIPA buffer (Thermo Fisher Scientific, cat# 89900) supplemented with protease inhibitor (Sigma-Aldrich, cat# 8820) and phosphatase inhibitor (Roche, cat# 04-906-845-001). Cell lysates were incubated on ice for 20 min, followed by centrifugation at 14,000

x g for 20 min at 4°C. The supernatant was collected, and protein concentrations were determined using the BCA Protein Assay Kit (Thermo Fisher Scientific, cat# 23225). Proteins were mixed with 4x Laemmli buffer (Bio-Rad, cat# 161-0747) containing dithiothreitol (DTT) and boiled at 95°C for 5 min to denature. Equal amounts of proteins from each sample were loaded onto 4-20% Mini-PROTEAN TGX Precast Gels (Bio-Rad, cat# 456-8093) and separated by electrophoresis in 1x Tris/Glycine/SDS running buffer (Bio-Rad, cat# 161-0732) at 100 V. Precision Plus Protein Dual Colour Standards (Bio-Rad, 161-0374) were included as molecular weight markers.

Proteins in gels were transferred onto PVDF membranes (Bio-Rad, cat# 1620177) using the Trans-Blot Turbo Transfer System (Bio-Rad, Hercules, USA), with a pre-programmed setting (25 V, 1 A, 30 min). After transfer, membranes were briefly washed in 1x Tris-buffered saline (TBS) (Bio-Rad, cat# 170-6435) and blocked with Western Blocker Solution (Sigma, cat# W0138-400ML) for 1 h at RT. Membranes were incubated overnight at 4°C with primary antibodies diluted in Western Blocker Solution. The following day, membranes were washed three times in TBS supplemented with 0.1% Tween-20 (TBST) and incubated with HRP-conjugated secondary antibodies for 1 h at RT. After washing with TBST, protein bands were detected using SuperSignal West Dura Extended Duration Substrate (Thermo Fisher Scientific, cat# 34075) and imaged on ChemiDoc Imaging System (Bio-Rad, Hercules, USA). Band intensities were quantified by ImageJ (NIH, Bethesda, USA) [31]. Protein expression levels were normalized against the loading control (β-Actin), and relative expressions were calculated. Antibodies used in Western blot analysis are listed in **ESM Table 21**.

Coculture experiments.

Cryovials containing BRI-4.13 (GAD65-specific, HLA-DRB1*04:01-restricted), BRI-5.325 (GAD65-specific, HLA-DRB1*04:04-restricted) and HA-specific (HLA-DRB1*04:04-restricted) $CD4^+$ T cells were thawed and resuspended in a medium consisting of RPMI 1640 (Gibco, cat#

11875093), 10% human heat inactivated serum (Millipore Sigma, cat# H3667), 1% Penicillin-Streptomycin (Gibco, cat# 15140122), and 1% L-Glutamine (Gibco, A2916801).

For some coculture experiments with BRI-4.13 CD4$^+$ T cells, T cells were thawed and rested overnight prior to coculture with peptide pulsed BSM or cytokine-treated ductal cells. In other coculture experiments with BRI-4.13 CD4$^+$ T cells, T cells were thawed and expanded in the presence of CD25 depleted, irradiated PBMCs with phytohemagglutinin (PHA) and 10 IU/ml of IL-2, as previously described [5-7] and then cultured for a minimum of 3-4 days without IL-2 to stabilize into resting state before coculture experiments.

For coculture experiments with BRI-5.325 CD4$^+$ T cells, T cells were thawed and rested overnight prior to coculture with ductal cells.

For coculture experiments with HA-specific (HLA-DRB1*04:04-restricted) CD4$^+$ T cells, T cells were thawed and expanded with CD25-depleted, irradiated PBMCs in the presence of phytohemagglutinin (PHA) and 10 IU/ml of IL-2, then cultured for 5 days without IL-2 to stabilize into a resting state before coculture experiments.

Ductal cells from donors with the HLA-DRB1*04:01 (for coculture with BRI-4.13) or HLA-DRB1*04:04 haplotype (for coculture with BRI-5.325 and HA-specific CD4$^+$ T cell clones) were used **(ESM Table 22)**. For cocultures with BRI-4.13 or BRI-5.325, control or cytokine-treated ductal cells were washed with PBS and then pulsed with GAD65 557I (NFIRMVISNPAAT) or Influenza HA306 peptide (PKYVKQNTLKLAT) at 50 µg/ml for 3 h at 37°C and 5% CO_2. For coculture with HA-specific CD4$^+$ T cell clone, cytokine-treated ductal cells were washed with PBS and then pulsed with GAD65 WT (NFFRMVISNPAAT) or Influenza HA306 peptide (PKYVKQNTLKLAT) at 50 µg/ml for 3 h at 37°C and 5% CO_2.

In parallel, for coculture with the BRI-4.13 clone, BSM cells were also pulsed with GAD65 557I (NFIRMVISNPAAT) or Influenza HA306 peptide (PKYVKQNTLKLAT) at 50 µg/ml in a T cell

medium for 3 h at 37°C and 5% CO_2. The T cell medium is composed of RPMI 1640 (Gibco, cat# 11875093), 10% human heat inactivated serum (Millipore Sigma, cat# H3667), 1% Penicillin-Streptomycin (Gibco, cat# 15140122), and 1% L-Glutamine (Gibco, cat# A2916801). Cells were then irradiated using Cesium at 15,000 rads or X-Ray at 40 Gy.

To track proliferation, BRI-4.13, BRI-5.325 or HA-specific $CD4^+$ T cells were stained with CellTrace CFSE (Thermo Fisher Scientific, cat# C34554), washed and resuspended in T cell medium prior to coculture experiments.

Finally, the BRI-4.13, BRI-5.325 or HA-specific $CD4^+$ T cells were added to peptide-pulsed ductal cells or BSM cells at a 1:5 ratio (100,000 T cell : 500,000 APC) or stimulated with plate-bound anti-CD3 antibodies at 1 μg/ml in 48-well plates, with a total volume of 1 ml per well. Cocultures were maintained for up to 5 days at 37°C and 5% CO_2.

Immunofluorescence staining.

Formalin-fixed paraffin-embedded (FFPE) pancreas tissues slides from non-diabetic donors were provided by the Southern California Islet Cell Resource Center (SC-IRC) at the City of Hope. Immunofluorescence staining was performed as previously described [9]. Slides were de-paraffinized by immersion in xylene for 15 min, rehydrated through graded ethanol washes, and subjected to antigen retrieval using Vector Antigen Unmasking Solution (Citric Acid Based, pH=5.5, Vector Laboratories, cat# H-3300) with the IHC-Tek Epitope Retrieval Steamer Set (IHCWORLD, cat# IW1102A) for 45 min. Slides were then washed with PBS supplemented with 0.1% Triton X-100 and permeabilized in PBS supplemented with 0.3% Triton X-100 for 30 min at RT. Next, samples were blocked with PBS supplemented with 10% donkey serum, 0.1% Triton X-100, and 1x Biogenex Laboratories Power Block (Thermo Fisher Scientific, cat# HK085-5K) for 2 h at RT. Slides were incubated with overnight at 4°C with primary antibodies, washed three times with PBS supplemented with 0.1% Triton X-100, and incubated with secondary antibodies

for 2 h at RT. Autofluorescence was minimized using the TrueView Quenching kit (Vector Laboratories, cat# SP-8400-15), followed by 10 min incubation with DAPI to stain nuclei. Vectashield vibrance antifade mounting medium (Vector Laboratories, cat# H-1700) was used to mount coverslips. Antibodies used in immunofluorescence staining are listed in **ESM Table 21**.

Representative images were acquired with Zeiss LSM 880 confocal microscope equipped with Plan-Apochromat 20x/0.8 or 40x/0.95 objectives. The pinhole was set to 1 Airy unit. Images were processed using Zeiss ZEN Blue or Black software (Carl Zeiss, Germany).

Whole-slide imaging of entire tissue sections was performed using a Zeiss Axioscan 7 slide scanner equipped with Plan-Apochromat 10x/0.45 or 20x/0.8 objectives for quantitative analysis. Images were analyzed using QuPath v4.2 [32]. Individual cells across the entire slide were identified using cell detection tool based on $DAPI^+$ nuclei. KRT19, GAD65/67 and CHGA positive cells were identified using object classification tool with respective makers. Thresholds for marker positivity were established using negative control images stained with only secondary antibodies.

Statistical analysis.

Statistical analyses were performed and plots generated using GraphPad Prism version 9.5 or 10. Results are expressed as mean ± SEM or mean ± SD as stated in the figure legends. Unless otherwise specified, significance between two groups were determined using unpaired two-tailed t-test and those between three or more groups were determined using ordinary one-way ANOVA. Statistical significance was defined as $p<0.05$. Significance levels were indicated as follows: *$p<0.05$, **$p<0.01$, ***$p<0.001$ and $p>0.05$, not significant (NS). Additional details regarding statistical tests are provided in the figure legends.

Supplemental Figures

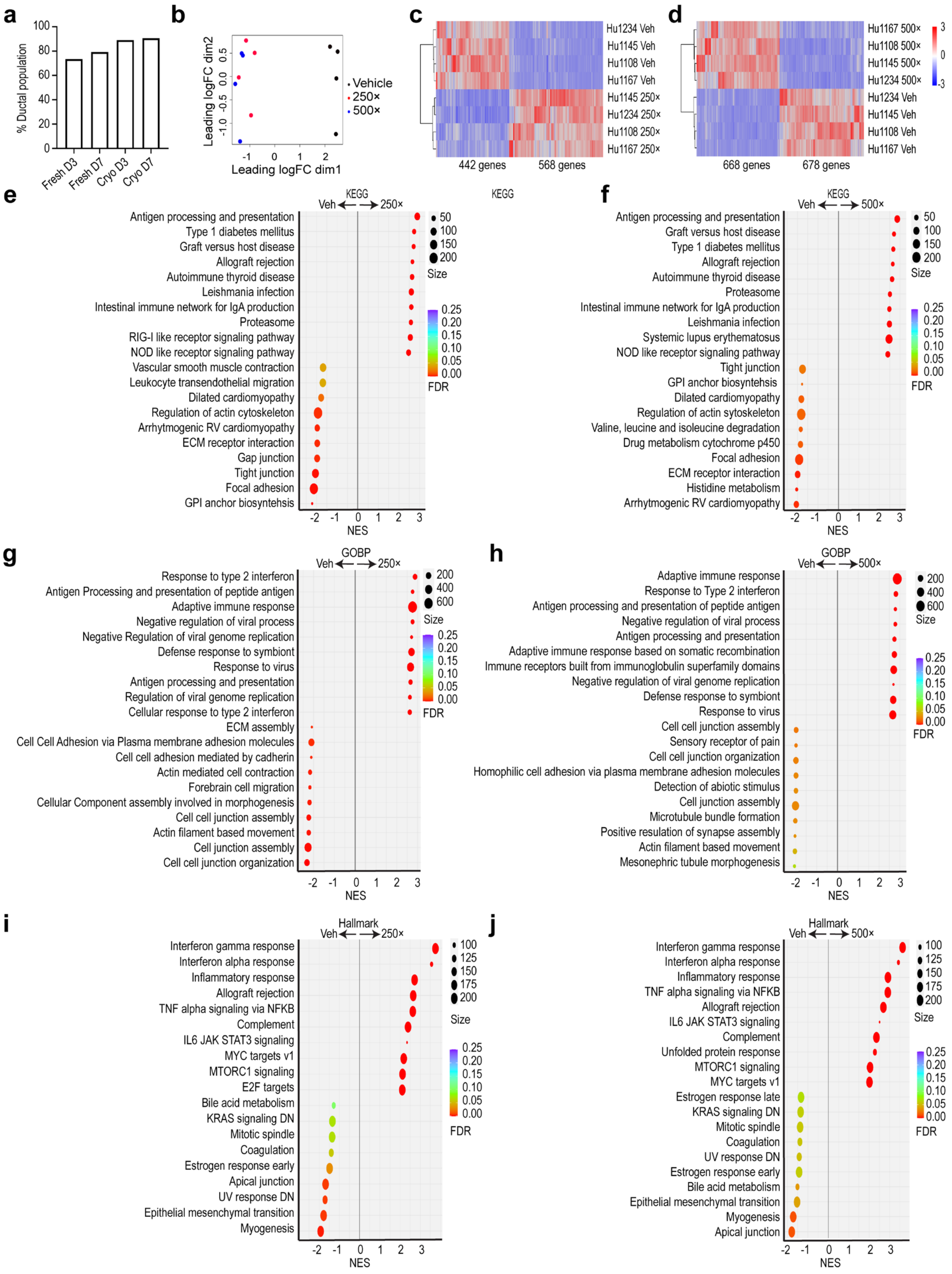

ESM Figure 1. Type 1 diabetes-associated proinflammatory cytokines induce gene pathways involved in "type 1 diabetes" and "antigen processing and presentation" in primary ductal cells from non-diabetic donors, related to Fig. 1.

(a) Data from Zook. et al. [2] were re-analyzed and presented. Pancreatic exocrine tissues were dissociated into single cells. Cells were immediately plated (fresh) or cryopreserved and thawed (Cryo) into a 3D suspension culture system. Cells from day 3 (D3) and day 7 (D7) in suspension culture were then subjected to single-cell RNA-seq analysis. Ductal population was defined as described by Zook. et al. [2]

(b) Bulk RNA-sequencing followed by principal components analysis of genes expressed in ductal cells treated with vehicle, 250× or 500× dose of cytokines. *N*=4 donors.

(c-d) Bulk RNAseq analysis from the current study. Unsupervised hierarchical clustering analysis of DEGs comparing 250× cytokines vs. vehicle **(c)**, and 500× cytokines vs. vehicle **(d)**. Colour intensity represents FPKM values that were normalized by subtracting the mean across samples and linearly scaled between -3 and 3. *N*=4 donors.

(e-j) Gene set enrichment analysis (GSEA) was conducted using the KEGG **(e-f)**, the GOBP **(g-h)**, and the Hallmark **(i-j)** molecular signature databases. Top biological pathways from 250× versus vehicle **(e,g,i)** and 500× versus vehicle **(f, h, j)** are shown. *N*=4 donors.

Abbreviations: DEGs, differentially expressed genes; FPKM, fragments per kilobase of transcript per million mapped reads; KEGG, Kyoto Encyclopedia of Genes and Genomes; GOBP, Gene Ontology Biological Process; NES, normalized expression score.

a

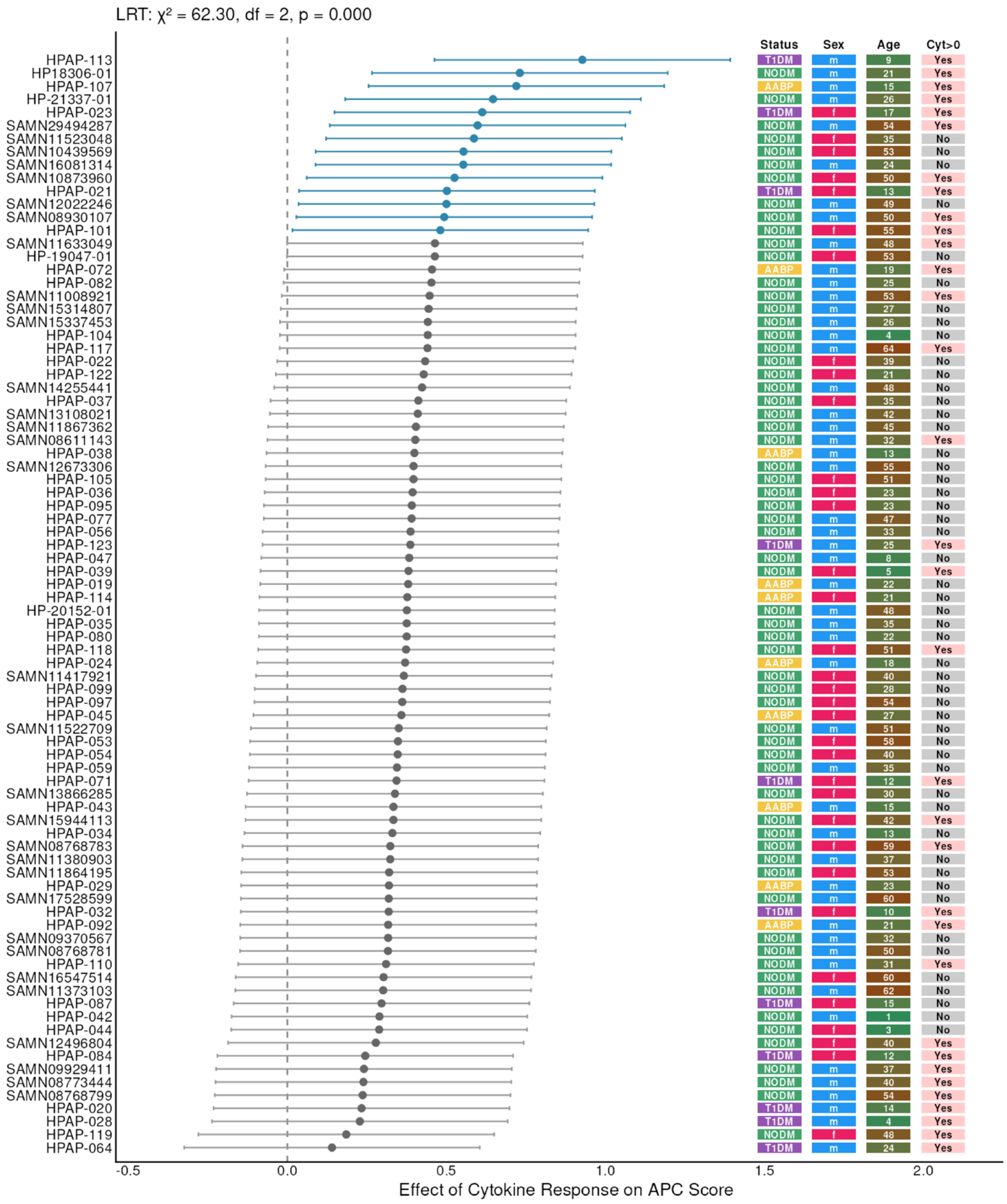

ESM Figure 2. Per-donor heterogeneity in the cytokine response-APC relationship, related to Fig 1.

(a) To test donor-level heterogeneity in the dose-response relationship, the random-intercepts model used for the positive component of the hurdle model was extended to a random-slopes

model, allowing the cytokine-APC relationship to vary by donor. The models were compared using a likelihood ratio test. The forest plot shows donor-specific slope estimates from the random-slopes linear mixed model (LMM). The model APC Score ~ Cytokine Response Score + (Cytokine Response Score | Donor) allows both intercepts and slopes to vary by donor. Each row represents one donor (*N*=84 donors with APC score >0). The positive component (Part 2) uses a stricter double-positive subset (APC>0 and cytokine>0, N=33 donors) **(Fig. 1k)**. Points indicate the estimated slope (effect of cytokine response on APC score) for each donor, with horizontal lines showing 95% confidence intervals. Blue indicates donors whose confidence interval does not cross zero (individually significant positive relationship), and grey indicates donors whose confidence interval crosses zero. All 84 donor slopes are positive, indicating a consistent direction of effect across the cohort. Fourteen donors show individually significant positive associations; significance reflects estimation precision rather than a distinct biological response class, as donors achieving significance have greater within-donor variance in cytokine response scores (mean range 0.18 vs 0.07), providing more statistical power to detect the relationship. Interaction effects between cytokine exposure and donor characteristics (age, diabetes status) were tested in both model components but were not significant (data not shown). Annotation columns show donor metadata: diabetes status (NODM, green; AABP, yellow; type 1 diabetes, purple), sex, age, and cytokine response status (score > 0). The likelihood ratio test (χ^2) indicates that slope magnitudes vary significantly across donors, reflecting quantitative heterogeneity in the strength of the cytokine–APC relationship.

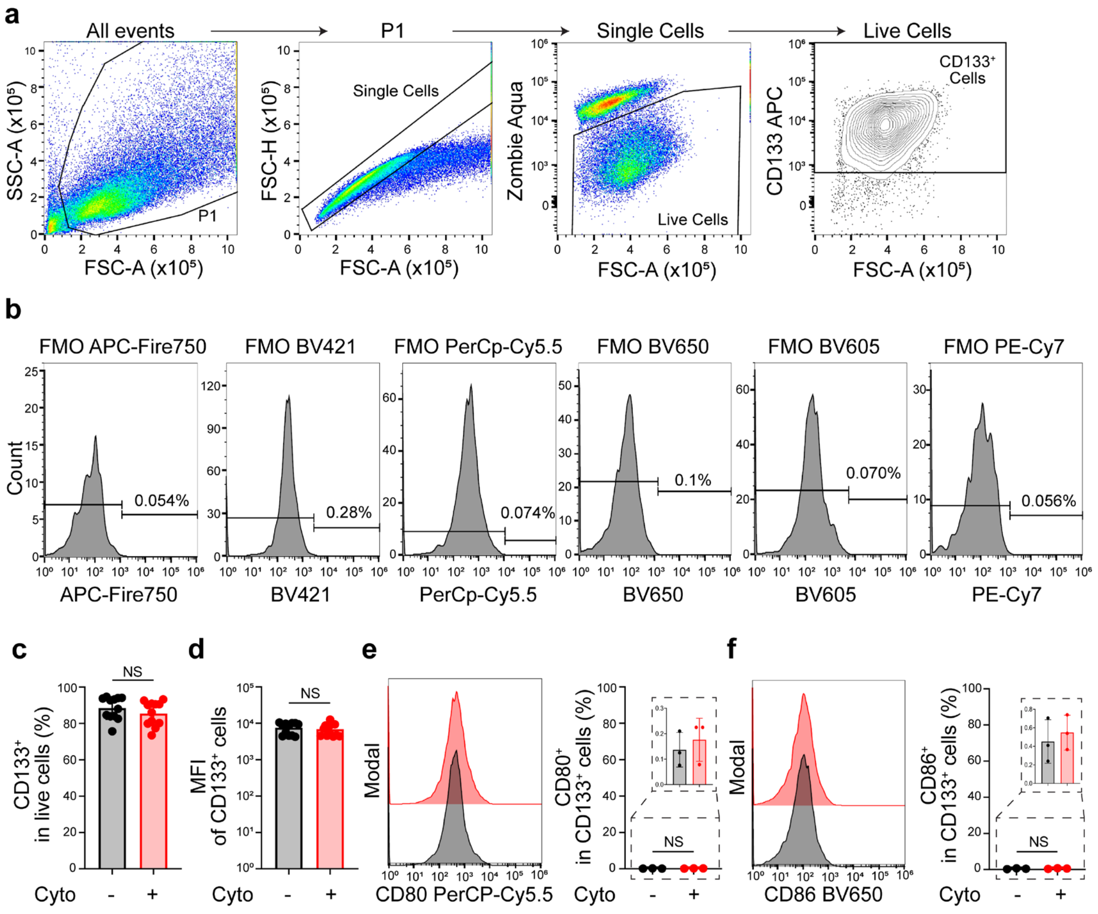

ESM Figure 3. Flow cytometry analysis of ductal cells, related to Fig. 2.

(a) Representative plots showing the sequential gating strategy of single live CD133+ ductal cells.

(b) Representative histograms for FMO controls. All gates were controlled using a stain that lacks just one of the fluorescence markers of interest.

(c-d) Quantification of percentage **(c)** or MFI **(d)** of CD133+ cells among single live cells. *N*=12 biological replicates from 11 unique donors. Each dot represents a biological replicate, with value as the mean of *n*=1-3 technical replicates. Data represent mean ± SD. Unpaired t-test was used to determine significance. NS: not significant (p>0.05).

(e-f) Representative histograms and quantification of percentage of CD80+ **(e)** or CD86+ cells **(f)** among CD133+ cells. *N*=3 donors. Each dot represents a donor. Data represent mean ± SD. Unpaired t-test was used to determine significance. NS: not significant (p>0.05).

Abbreviations: FMO, fluorescence minus one; MFI, mean fluorescence intensity; SSC-A, side scatter area; FSC-A, forward scatter area; APC, Allophycocyanin; BV, Brilliant Violet; PE, Phycoerythrin; PerCp, Peridinin-Chlorophyll-Protein; P1, population 1.

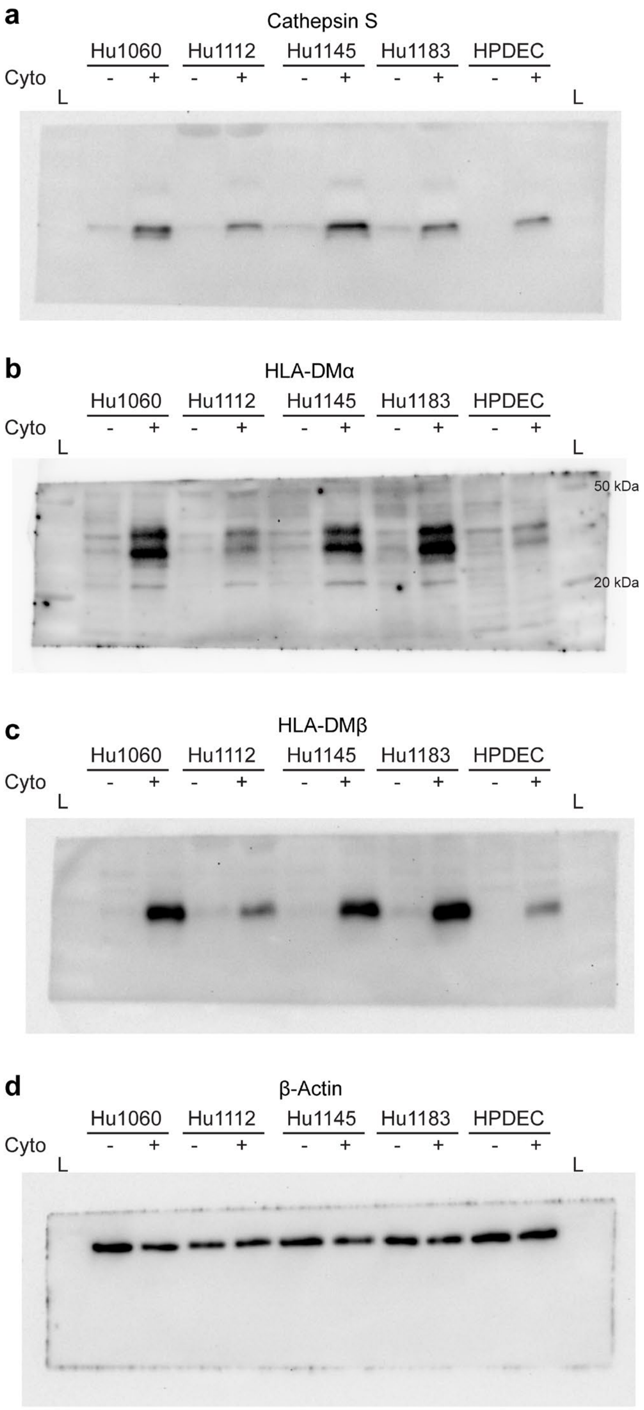

ESM Figure 4. Full-sized Western blot images, related to Fig. 2.

Uncropped images for Cathepsin S **(a)**, HLA-DMα **(b)**, HLA-DMβ **(c)** and β-Actin **(d)** are shown. Ladder (L): Precision Plus Protein Dual Colour Standards (Bio-Rad, 1610374). *N*=4 donors.

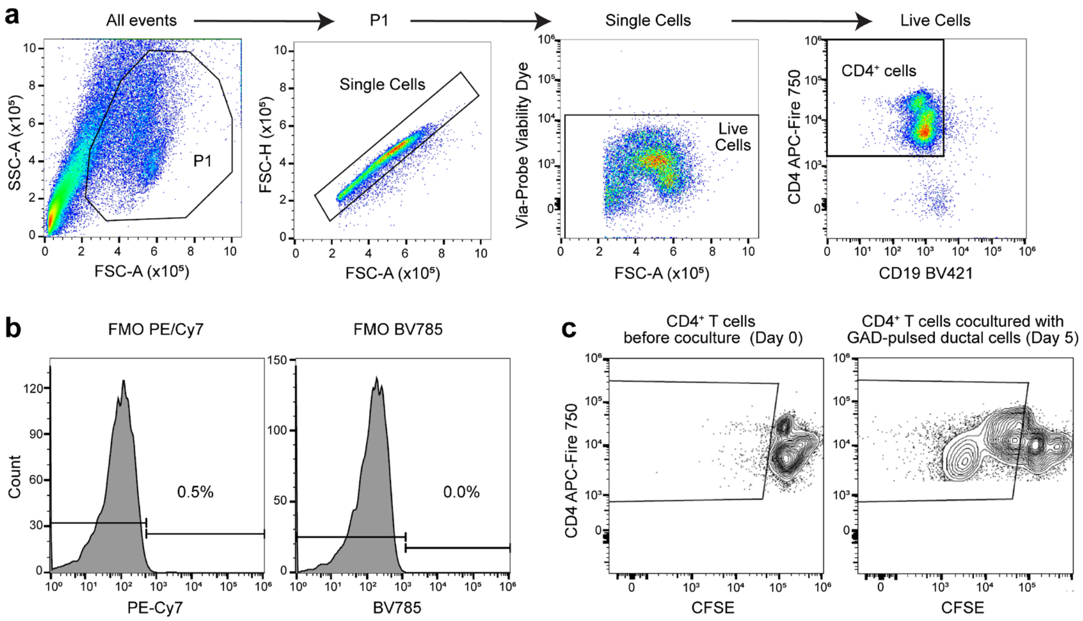

ESM Figure 5. Gating strategy of CD4⁺ T cells in coculture experiments, related to Fig. 3 and 4.

(a) Representative plots showing sequential gating of single live CD4⁺ T cells.

(b) Representative histograms for FMO controls. All gates were controlled using a stain that lacks just one of the fluorescence markers of interest.

(c) Representative gating for CFSE-diluted cells.

Abbreviations: FMO, fluorescence minus one; CFSE, carboxyfluorescein succinimidyl ester; GAD, glutamate decarboxylase; SSC-A, side scatter area; FSC-A, forward scatter-area; BV, Brilliant Violet; PE/Cy7, Phycoerythrin-Cyanine 7; P1, population 1.

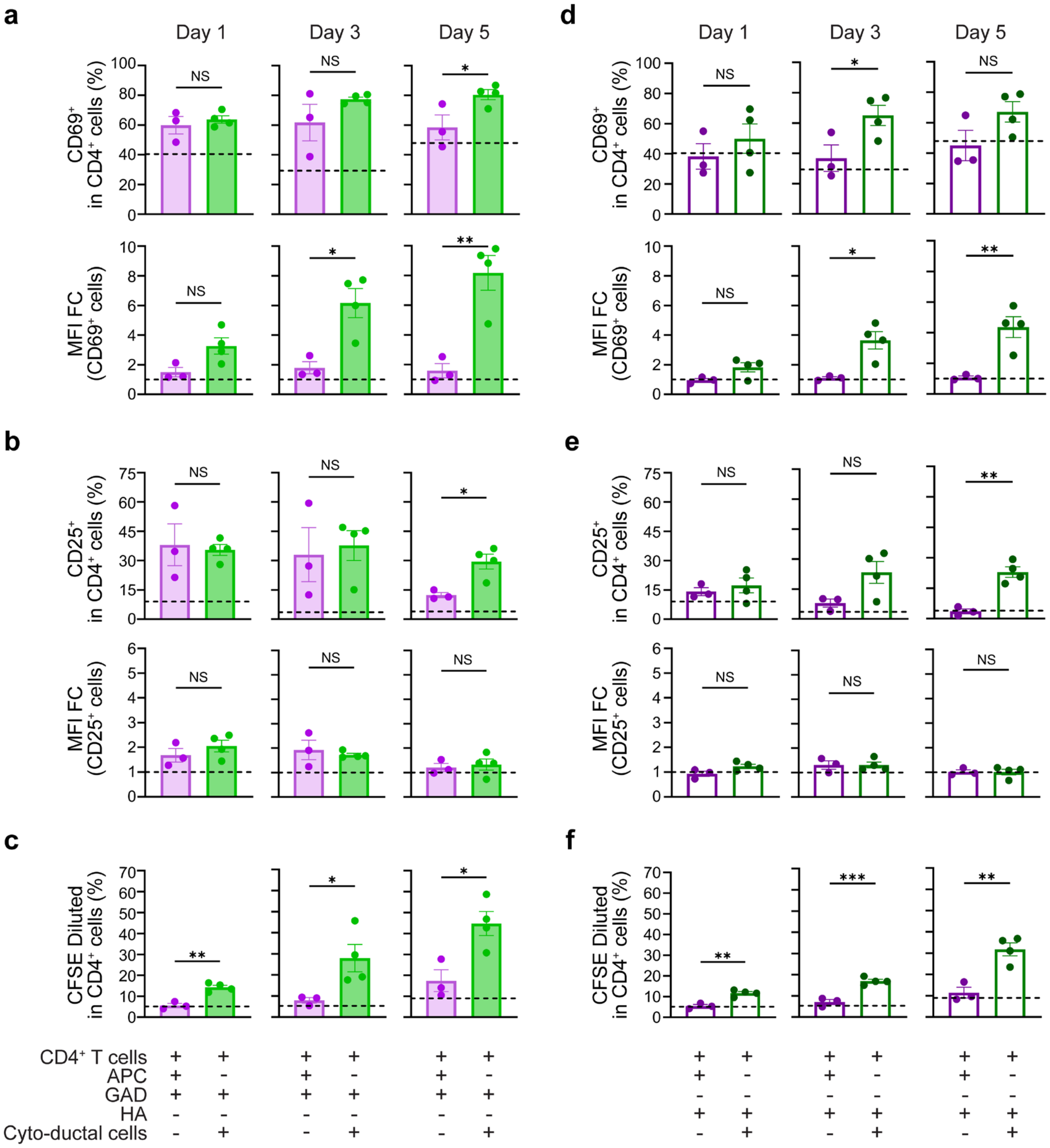

ESM Figure 6. Cytokine-treated ductal cells are equal or more effective than BSM cells in inducing activation and proliferation of BRI-4.13 CD4⁺ T cell clone, related to Fig. 3 and 4. From the same dataset shown in **Fig. 3 and 4**, results on BRI-4.13 CD4⁺ T cells cocultured with BSM APCs (purple) or cytokine-treated ductal cells (green) were selected and compared. Cytokine-treated ductal and BSM cells were pulsed with a peptide from GAD (filled bars) **(a-c)** or

HA (open bars) **(d-f)**. End points were analyzed at days 1, 3 and 5 post-coculture as shown in the left, middle, and right panels, respectively. *N*=3-4 biological replicates of T cells that were cocultured with ductal cells from 3 unique donors. Each dot represents a biological replicate, with value as the mean of *n*=2 technical replicates. Data represent mean ± SEM. The dashed horizontal lines indicate the mean value of the $CD4^+$ T cells alone group from **Fig. 3 and 4**. The fold change of MFI was relative to $CD4^+$ T cells alone group. Unpaired t-test was used for statistical analysis, with significance indicated as *p<0.05, **p<0.01, ***p<0.001 and p>0.05, not significant (NS).

Abbreviations: APC, antigen presenting cell; FC: fold change; GAD, glutamate decarboxylase; HA, hemagglutinin; MFI, mean fluorescence intensity; CFSE, carboxyfluorescein succinimidyl ester.

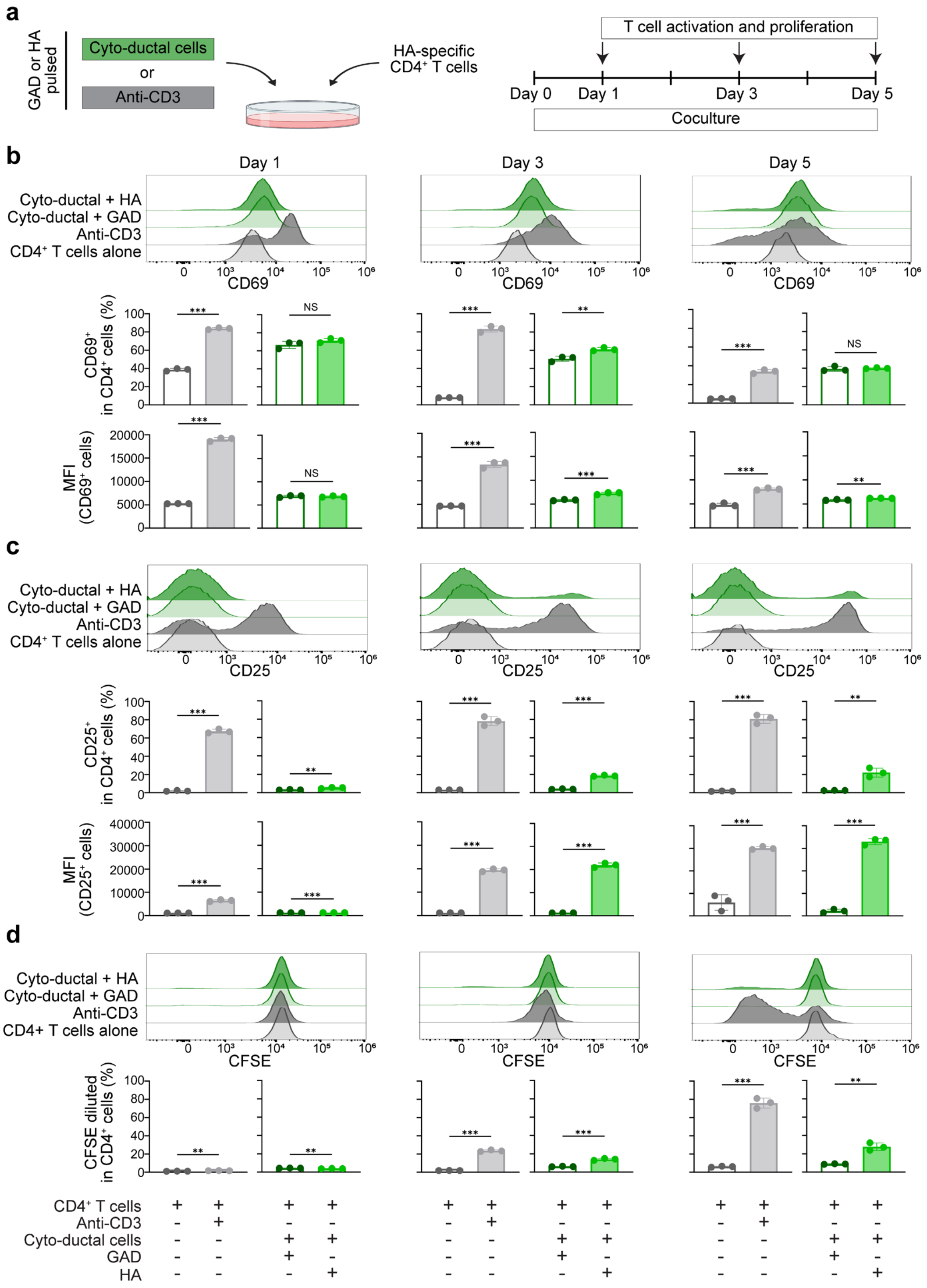

a
GAD or HA pulsed
Cyto-ductal cells
or
Anti-CD3
HA-specific CD4⁺ T cells
T cell activation and proliferation
Day 0
Day 1
Day 3
Day 5
Coculture
b
Day 1
Day 3
Day 5
Cyto-ductal + HA
Cyto-ductal + GAD
Anti-CD3
CD4⁺ T cells alone
CD69
CD69⁺ in CD4⁺ cells (%)
MFI (CD69⁺ cells)
NS
c
CD25
CD25⁺ in CD4⁺ cells (%)
MFI (CD25⁺ cells)
d
CD4+ T cells alone
CFSE
CFSE diluted in CD4⁺ cells (%)
CD4⁺ T cells
Anti-CD3
Cyto-ductal cells
GAD
HA

ESM Figure 7. Cytokine-treated ductal cells pulsed with HA peptide stimulate activation and proliferation of HA-specific $CD4^+$ T cells.

(a) Experimental workflow for the activation and proliferation assay of HA-specific (HLA-DRB1*04:04-restricted) $CD4^+$ T cells. Created with BioRender.com. Erdem, N. (2026) https://BioRender.com/89xxni2

(b-c) Top, middle and lower panels indicate representative histograms, percentage of $CD4^+$ cells expressing the designated marker, and the MFI of the designated marker, respectively. Colour scheme indicates cultured HA-specific $CD4^+$ T cells alone (open grey), stimulated with anti-CD3 (filled grey), and cocultured with GAD65-pulsed (GAD65 WT, NFFRMVISNPAAT, open green) or HA-pulsed cytokine-treated ductal cells (HA306 peptide, PKYVKQNTLKLAT, filled green). End points were analyzed at days 1, 3 and 5 post-coculture as shown in the left, middle, and right panels, respectively. n=3 technical replicates of $CD4^+$ T cells cocultured with ductal cells from 1 unique donor. Data represent mean ± SD. Unpaired t-test was used for statistical analysis. Significance is indicated as *p<0.05, **p<0.01, ***p<0.001.

Abbreviations: GAD, glutamate decarboxylase; HA, hemagglutinin; MFI, mean fluorescence intensity

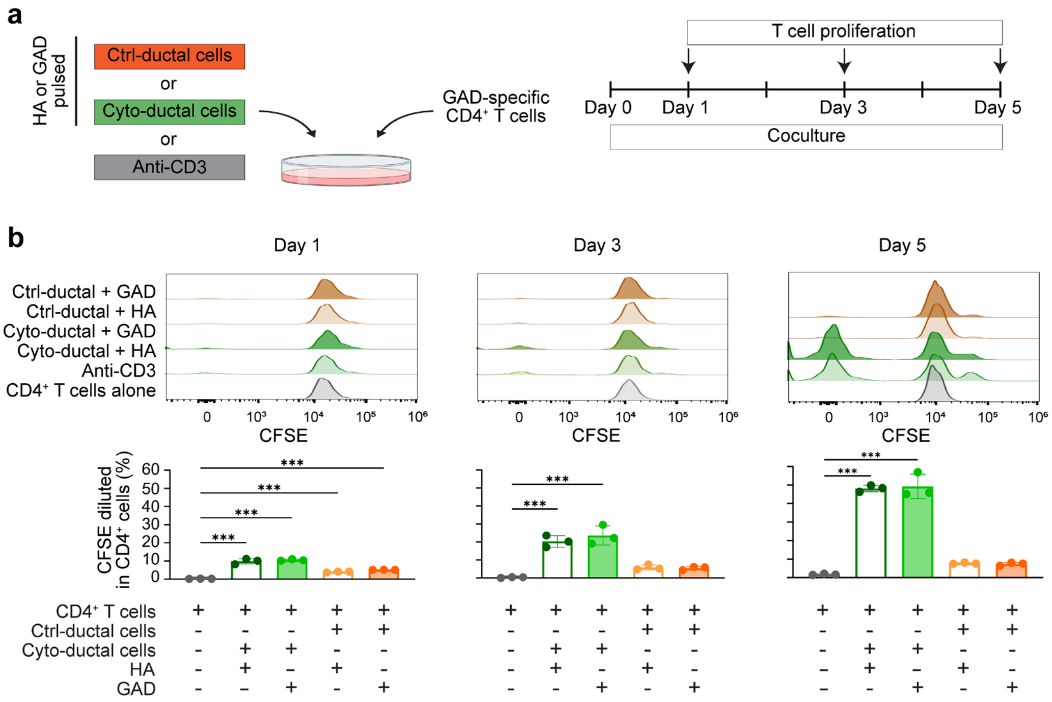

ESM Figure 8. Control ductal cells do not increase the proliferation of GAD65-specific CD4⁺ T cells at days 3 and 5.

(a) Experimental workflow for the proliferation assay of BRI-5.325 CD4⁺ T cells. Created with BioRender.com. Erdem, N. (2026) https://BioRender.com/v6e6fmw

(b-c) Representative histograms and quantification of percentage of CFSE-diluted CD4⁺ T cells. Colour scheme indicates cultured BRI-5.325 T cells alone (open grey), cocultured with HA-pulsed (HA306 peptide, PKYVKQNTLKLAT, open green) or GAD65-pulsed cytokine-treated ductal cells (GAD65 557I, NFIRMVISNPAAT, filled green), and cocultured with HA-pulsed (HA306 peptide, PKYVKQNTLKLAT, open orange) or GAD65-pulsed control ductal cells (GAD65 557I, NFIRMVISNPAAT, filled orange). End points were analyzed at days 1, 3 and 5 post-coculture as shown in the left, middle, and right panels, respectively. *n*=3 technical replicates of CD4⁺ T cells

cocultured with ductal cells from 1 unique donor. Data represent mean ± SD. Unpaired t-test was used for statistical analysis. Significance is indicated as *$p<0.05$, **$p<0.01$, ***$p<0.001$.

Abbreviations: GAD, glutamate decarboxylase; HA, hemagglutinin; CFSE: carboxyfluorescein succinimidyl ester

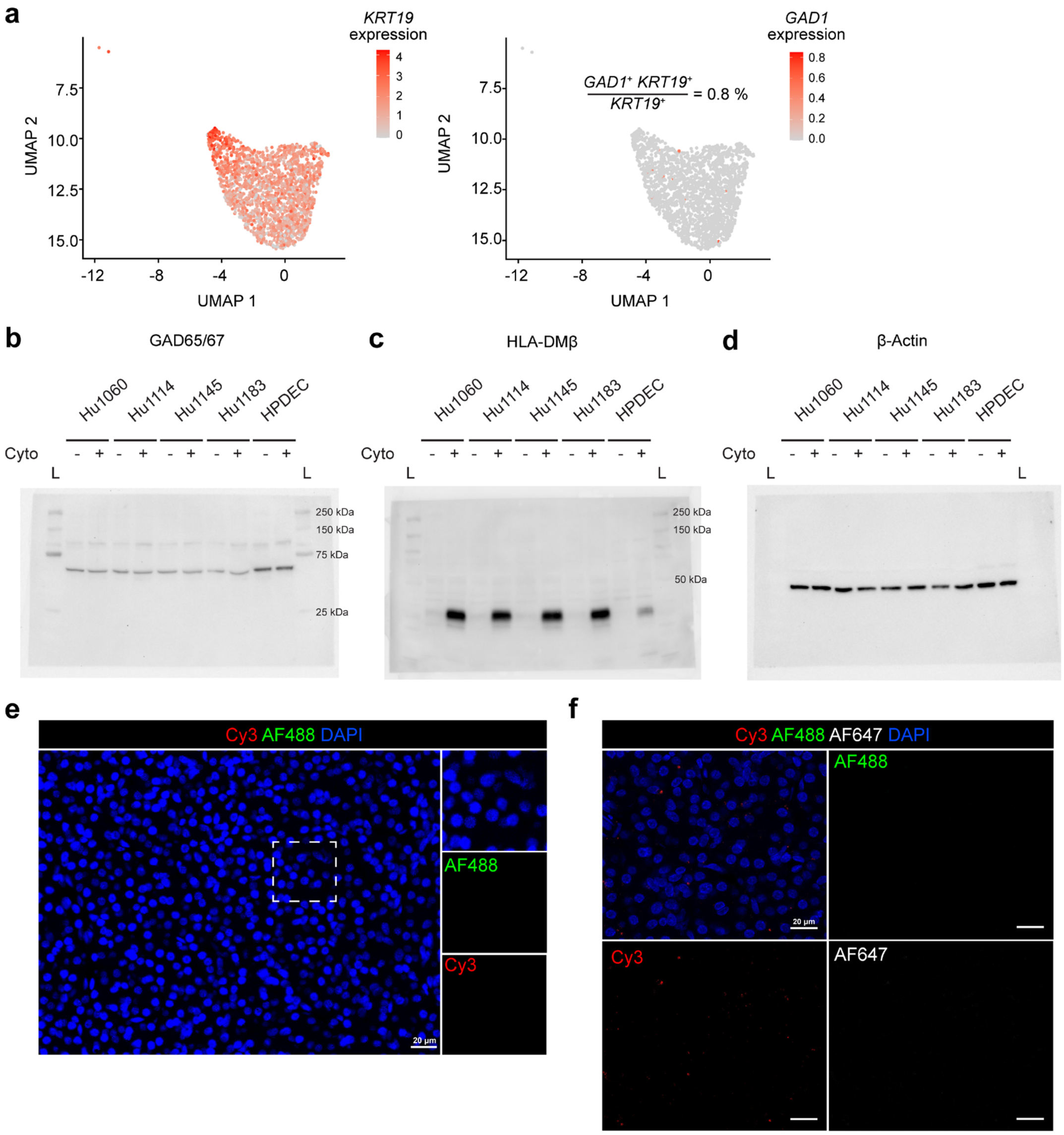

ESM Figure 9. Some pancreatic ductal cells express GAD1 mRNA and GAD65/67 protein, related to Fig. 5.

(a) Data from Zook. et al. [2] were re-analyzed and presented. After islet isolation from a donor pancreas, the exocrine tissue was dissociated into single cells and immediately processed for single-cell RNAseq analysis. Only the ductal cell population based on Zook. et al. [2] was selected

for the UMAP plots. Expression of *KRT19* (left panel) and *GAD1* (right panel) are shown. Each dot represents a single cell.

(b-d) Uncropped Western blot images for GAD65/67 **(b)**, HLA-DMβ **(c)** or β-Actin **(d)**, related to **Fig. 5a**. Ladder (L): Precision Plus Protein Dual Colour Standards (Bio-Rad, 1610374). *N*=4 donors.

(e) A representative image of a negative control (secondary antibodies only) for co-immunofluorescence staining experiment, related to **Fig. 5c**. Negative control slides were imaged on the same day as those stained with antigen-specific antibodies, and the images were analyzed and adjusted identically. Experiments from all donors included both negative control staining and the staining with antigen-specific antibodies. Scale bars, 20 μm.

(f) A representative image of a negative control (secondary antibodies only) for co-immunofluorescence staining experiment, related to **Fig. 5e**. Negative control slides were imaged in the same day as those stained with antigen-specific antibodies, and the images were analyzed and adjusted identically. Experiments from all donors included both negative control staining and the staining with antigen-specific antibodies. Scale bars, 20 μm

Abbreviations: UMAP, Uniform Manifold Approximation and Projection; FFPE, formalin-fixed paraffin-embedded; Cy3, cyanine 3, AF488, Alexa Fluor 488; AF647, Alexa Fluor 647; DAPI, 4′,6-diamidino-2-phenylindole.

References

[1] Qi M, Valiente L, McFadden B, et al. (2015) The Choice of Enzyme for Human Pancreas Digestion is a Critical Factor for Increasing the Success of Islet Isolation. Transplantation direct 1(4). 10.1097/TXD.0000000000000522

[2] Zook HN, Quijano JC, Ortiz JA, et al. (2024) Activation of ductal progenitor-like cells from adult human pancreas requires extracellular matrix protein signaling. iScience 27(3): 109237. 10.1016/j.isci.2024.109237

[3] Zook HN, Quijano JC, Ortiz JA, Donohue C, Erdem N, Ku HT (2025) Protocol to study ductal progenitor-like cells from the adult human pancreas using 3D suspension and methylcellulose-based culture systems. STAR Protoc 6(2): 103847. 10.1016/j.xpro.2025.103847

[4] Quijano JC, Wedeken L, Ortiz JA, et al. (2023) Methylcellulose colony assay and single-cell micro-manipulation reveal progenitor-like cells in adult human pancreatic ducts. Stem Cell Reports 18(3): 618-635. 10.1016/j.stemcr.2023.02.001

[5] Reijonen H, Novak EJ, Kochik S, et al. (2002) Detection of GAD65-specific T-cells by major histocompatibility complex class II tetramers in type 1 diabetic patients and at-risk subjects. Diabetes 51(5): 1375-1382. 10.2337/diabetes.51.5.1375

[6] Matthis J, King V, Reijonen H (2019) Production of Antigen-Specific Human $CD4^{+}$ T Cell Lines and Clones. Methods in molecular biology (Clifton, NJ) 1988: 387-402. 10.1007/978-1-4939-9450-2_27

[7] Reijonen H, Mallone R, Heninger AK, et al. (2004) GAD65-specific $CD4^{+}$ T-cells with high antigen avidity are prevalent in peripheral blood of patients with type 1 diabetes. Diabetes 53(8): 1987-1994. 10.2337/diabetes.53.8.1987

[8] Reijonen H, Elliott JF, van Endert P, Nepom G (1999) Differential Presentation of Glutamic Acid Decarboxylase 65 (GAD65) T Cell Epitopes Among HLA-DRB1*0401-Positive Individuals. The Journal of Immunology 163(3): 1674-1681. 10.4049/jimmunol.163.3.1674

[9] Ortiz JA, Ghazalli N, Lopez K, et al. (2024) Trefoil Factor 2 Expressed by the Murine Pancreatic Acinar Cells Is Required for the Development of Islets and for beta-Cell Function During Aging. Diabetes 73(9): 1447-1461. 10.2337/db23-0490

[10] Bolger AM, Lohse M, Usadel B (2014) Trimmomatic: a flexible trimmer for Illumina sequence data. Bioinformatics (Oxford, England) 30(15): 2114-2120. 10.1093/bioinformatics/btu170

[11] Chen S, Zhou Y, Chen Y, Gu J (2018) fastp: an ultra-fast all-in-one FASTQ preprocessor. Bioinformatics (Oxford, England) 34(17): i884-i890. 10.1093/bioinformatics/bty560

[12] Dobin A, Davis CA, Schlesinger F, et al. (2013) STAR: ultrafast universal RNA-seq aligner. Bioinformatics (Oxford, England) 29(1): 15-21. 10.1093/bioinformatics/bts635

[13] Anders S, Huber W (2010) Differential expression analysis for sequence count data. Genome Biol 11(10): R106. 10.1186/gb-2010-11-10-r106

[14] Robinson MD, McCarthy DJ, Smyth GK (2010) edgeR: a Bioconductor package for differential expression analysis of digital gene expression data. Bioinformatics (Oxford, England) 26(1): 139-140. 10.1093/bioinformatics/btp616

[15] Wu T, Hu E, Xu S, et al. (2021) clusterProfiler 4.0: A universal enrichment tool for interpreting omics data. Innovation (Camb) 2(3): 100141. 10.1016/j.xinn.2021.100141

[16] Xu S, Hu E, Cai Y, et al. (2024) Using clusterProfiler to characterize multiomics data. Nat Protoc 19(11): 3292-3320. 10.1038/s41596-024-01020-z

[17] Yu G (2024) Thirteen years of clusterProfiler. Innovation (Camb) 5(6): 100722. 10.1016/j.xinn.2024.100722

[18] Yu G, Wang LG, Han Y, He QY (2012) clusterProfiler: an R package for comparing biological themes among gene clusters. Omics : a journal of integrative biology 16(5): 284-287. 10.1089/omi.2011.0118

[19] Consortium P PanKBase Single-Cell RNA Sequencing Data Repository. Available from https://pankbase-data-v1.s3.us-west-

2.amazonaws.com/analysis_resources/single_cell_objects/060425_scRNA_v3.3.rds. Accessed 2 December 2025

[20] Vu HTH, Sun HAN, Sharp S, et al. (2025) 2121-LB: PanKbase Integrated Single-Cell Map—A Comprehensive Atlas of Human Pancreatic Islets Unlocking Insights into Type 1 and Type 2 Diabetes. Diabetes 74(Supplement_1): 2121-LB. 10.2337/db25-2121-LB

[21] Fasolino M, Schwartz GW, Patil AR, et al. (2022) Single-cell multi-omics analysis of human pancreatic islets reveals novel cellular states in type 1 diabetes. Nat Metab 4(2): 284-299. 10.1038/s42255-022-00531-x

[22] Kaestner KH, Powers AC, Naji A, Atkinson MA (2019) NIH Initiative to Improve Understanding of the Pancreas, Islet, and Autoimmunity in Type 1 Diabetes: The Human Pancreas Analysis Program (HPAP). Diabetes 68(7): 1394-1402. 10.2337/db19-0058

[23] Shapira SN, Naji A, Atkinson MA, Powers AC, Kaestner KH (2022) Understanding islet dysfunction in type 2 diabetes through multidimensional pancreatic phenotyping: The Human Pancreas Analysis Program. Cell Metab 34(12): 1906-1913. 10.1016/j.cmet.2022.09.013

[24] Patil AR, Schug J, Naji A, Kaestner KH, Faryabi RB, Vahedi G (2023) Single-cell expression profiling of islets generated by the Human Pancreas Analysis Program. Nat Metab 5(5): 713-715. 10.1038/s42255-023-00806-x

[25] Andreatta M, Carmona SJ (2021) UCell: Robust and scalable single-cell gene signature scoring. Comput Struct Biotechnol J 19: 3796-3798. 10.1016/j.csbj.2021.06.043

[26] Hao Y, Stuart T, Kowalski MH, et al. (2024) Dictionary learning for integrative, multimodal and scalable single-cell analysis. Nat Biotechnol 42(2): 293-304. 10.1038/s41587-023-01767-y

[27] Brooks ME, Kristensen K, van Benthem KJ, et al. (2017) glmmTMB Balances Speed and Flexibility Among Packages for Zero-inflated Generalized Linear Mixed Modeling. R J 9(2): 378-400. Doi 10.32614/Rj-2017-066

[28] Bates D, Mächler M, Bolker BM, Walker SC (2015) Fitting Linear Mixed-Effects Models Using lme4. J Stat Softw 67(1): 1-48. DOI 10.18637/jss.v067.i01

[29] Kuznetsova A, Brockhoff PB, Christensen RHB (2017) lmerTest Package: Tests in Linear Mixed Effects Models. J Stat Softw 82(13): 1-26. DOI 10.18637/jss.v082.i13

[30] Landau W (2021) The targets R package: a dynamic Make-like function-oriented pipeline toolkit for reproducibility and high-performance computing. Journal of Open Source Software 6(57): 2959. 10.21105/joss.02959

[31] Schneider CA, Rasband WS, Eliceiri KW (2012) NIH Image to ImageJ: 25 years of image analysis. Nature methods 9(7): 671-675. 10.1038/nmeth.2089

[32] Bankhead P, Loughrey MB, Fernandez JA, et al. (2017) QuPath: Open source software for digital pathology image analysis. Sci Rep 7(1): 16878. 10.1038/s41598-017-17204-5
